# Point prevalence of evidence-based antimicrobial use among hospitalized patients in sub-Saharan Africa: a systematic review and meta-analysis

**DOI:** 10.1038/s41598-024-62651-6

**Published:** 2024-06-02

**Authors:** Minyahil Tadesse Boltena, Mirkuzie Wolde, Belachew Hailu, Ziad El-Khatib, Veronika Steck, Selam Woldegerima, Yibeltal Siraneh, Sudhakar Morankar

**Affiliations:** 1 Ethiopian Evidence Based Health Care Centre: A Joanna Briggs Institute’s Center of Excellence, Faculty of Public Health, Institute of Health, Jimma University, Jimma, Ethiopia; 2 Armauer Hansen Research Institute, Ministry of Health, Addis Ababa, Ethiopia; 3 University of Technology Sydney, Sydney, Australia; 4 Global Public Health Department, Karolinska Institute, Solna, Sweden; 5 Department of Pharmacology and Therapeutics, Faculty of Life Sciences, McGill University, Montreal, Canada; 6 Nuffield Department of Population Health, University of Oxford, Oxford, UK

**Keywords:** Antibiotic prescribing, Antimicrobial use, Evidence-based healthcare, Hospitalized patients, Point prevalence survey, Sub-Saharan Africa, Health care, Medical research

## Abstract

Excessive and improper use of antibiotics causes antimicrobial resistance which is a major threat to global health security. Hospitals in sub-Saharan Africa (SSA) has the highest prevalence of antibiotic use. This systematic review and meta-analysis aimed to determine the pooled point prevalence (PPP) of evidence-based antimicrobial use among hospitalized patients in SSA. Literature was retrieved from CINAHL, EMBASE, Google Scholar, PubMed, Scopus, and Web of Science databases. Meta-analysis was conducted using STATA version 17. Forest plots using the random-effect model were used to present the findings. The heterogeneity and publication bias were assessed using the *I*^*2*^ statistics and Egger’s test. The protocol was registered in PROSPERO with code CRD42023404075. The review was conducted according to PRISMA guidelines. A total of 26, 272 study participants reported by twenty-eight studies published from 10 countries in SSA were included. The pooled point prevalence of antimicrobial use in SSA were 64%. The pooled estimate of hospital wards with the highest antibiotic use were intensive care unit (89%). The pooled prevalence of the most common clinical indication for antibiotic use were community acquired infection (41%). The pooled point prevalence of antimicrobial use among hospitalized patients were higher in SSA. Higher use of antibiotics was recorded in intensive care units. Community acquired infection were most common clinical case among hospitalized patients. Health systems in SSA must design innovative digital health interventions to optimize clinicians adhere to evidence-based prescribing guidelines and improve antimicrobial stewardship.

## Introduction

Global antibiotic consumption rates surged by 46%, indicating that the defined daily dose (DDD) per 1000 population per day rose from 9.8 to 14.3 between 2000 and 2018^1^. In low- and middle-income countries (LMICs), antibiotic usage increased by 76% and is projected to continue rising by 2030^2^. Hospitals in SSA have a higher prevalence of antibiotic usage (50%), including the use of broad-spectrum cephalosporins and penicillin^3^.

With improving economies and enhanced access to pharmaceuticals, many of LMICs now revealed antibiotic consumption rates comparable to or even surpassing those of high-income countries^4^. Sub-Saharan African countries are experiencing a similar trend in antibiotic consumption, which could be exacerbated by the region’s exceptionally high infectious disease burden^5^. This sharp rise in antibiotic usage with or without prescription, has become a pressing public health concern due to its strong association with the development of antimicrobial resistance in low resource clinical context^6,7^.

The misuse and overuse of antibiotics have led to increased rates of antimicrobial resistance, higher levels of morbidity and mortality, and escalated healthcare costs in low-income countries^8,9^. To address this issue, evaluating antibiotic prescribing patterns among patients in healthcare facilities is essential in identifying opportunities for antimicrobial stewardship to promote appropriate antibiotic use^10,11^.

Point prevalence studies have proven to be reliable and valid methods for measuring antibiotic use among hospitalized patients^12^. They provide crucial insights into the current state of antibiotic use within healthcare settings, aiding in the identification of patterns and deviations from recommended practices^13^. This data can inform targeted interventions to improve guideline adherence, optimize antibiotic selection, dosing, and duration, and reduce inappropriate prescriptions^14,15^. By promoting evidence-based clinical decisions, these studies contribute to the prevention of antibiotic overuse, the emergence of antimicrobial resistance, and the enhancement of patient outcomes, thus serving as a vital tool in advancing the quality and effectiveness of real-world healthcare practices^16,17^.

In sub-Saharan Africa, several point prevalence studies have reported a high rate of antibiotic use among hospitalized patients, along with inappropriate usage in healthcare facilities^18^. However, there is limited regional-level data available to describe the point prevalence of antibiotic use among hospitalized patients in SSA^19^. Understanding the epidemiology of antibiotic use in this context and assessing the quality of antibiotic prescribing are critical steps in designing effective antimicrobial stewardship interventions aimed at encouraging the rational use of antibiotics and improving clinical outcomes for patients^20^. Therefore, this systematic review and meta-analysis aimed to determine the pooled point prevalence of antibiotic use among hospitalized patients in sub-Saharan Africa.

## Methods

### Search strategy and selection of studies

**Figure 1:**
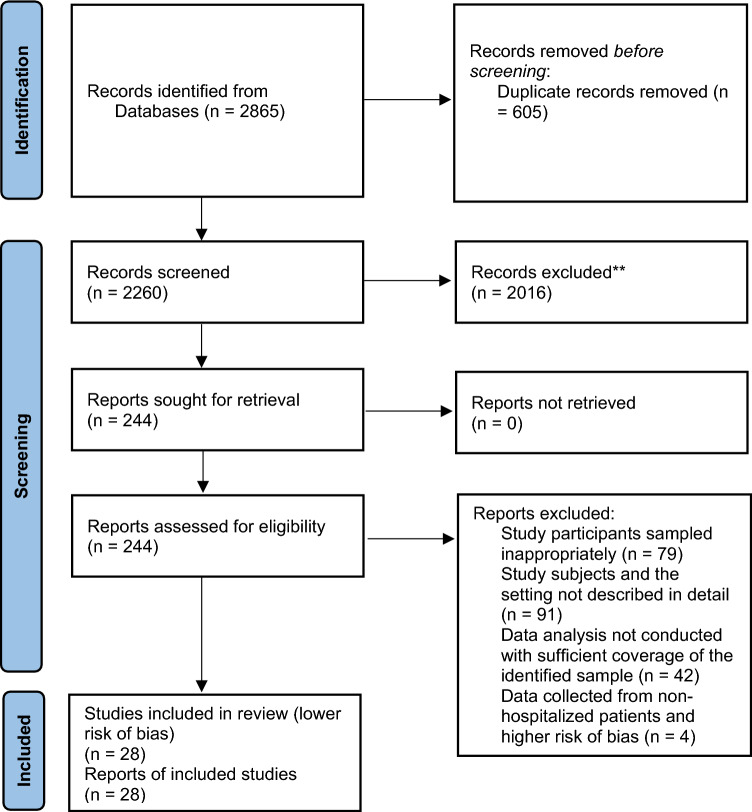
PRISMA flow diagram of included studies: Page MJ, McKenzie JE, Bossuyt PM, Boutron I, Hoffmann TC, Mulrow CD, et al. The PRISMA 2020 statement: an updated guideline for reporting systematic reviews. BMJ 2021;372:n71. 10.1136/bmj.n71.

The search strategy aimed to find both published and unpublished literature. Initially, a preliminary search was conducted on the Google Scholar to identify indexed full texts or metadata of scholarly literature on the topic. We adapted key terms as needed for each database, utilizing a combination of MeSH terms and text words, employing Boolean operators “AND” and “OR” for searches in databases like CINAHL, PubMed, EMBASE, Scopus, and Web of Science (Appendix I). Additionally, we examined the reference lists of selected studies for potential additional sources. No restrictions were imposed based on language or publication year. After the search, all identified citations were organized and imported into EndNote version 15.0, with duplicates removed. Two independent reviewers (MTB and BH) screened titles and abstracts, and a third reviewer (ZEK) cross-checked them against the inclusion and exclusion criteria. Relevant studies meeting the criteria were obtained in full, along with their citation details. Studies reporting the point prevalence of antibiotic use among hospitalized patients in SSA, which were published from 2013 to 2023 were eligible for inclusion. Excluded were systematic reviews, Studies having participants sampled inappropriately and the setting not described in detail studies, data analysis not conducted with sufficient coverage of the identified sample, and literature from high-income countries. Two independent reviewers (MTB and BH) assessed the full text of selected citations against the inclusion criteria, with a third reviewer (LWT) conducting a double-check. Reasons for excluding studies failing to meet the inclusion criteria upon full text review were documented. Any disagreements between reviewers at each stage of the study selection process were resolved through discussion or by consulting a third reviewer. The PRISMA checklist (Appendix II) and flow chart was used to describe the matching pages in the manuscript with the number of articles identified, included, and excluded with justifications. The results of the search were fully reported in the final systematic review and presented in a Preferred Reporting Items for Systematic Reviews and Meta-analyses (PRISMA) flow diagram (Fig. 1)^21^.

### Operational definitions

#### Point prevalence survey of antimicrobial use

Is a structured assessment done in healthcare settings to determine the percentage of patients receiving antimicrobial treatment at a particular moment^22^. Its goal is to assess the appropriateness of antimicrobial use, including choice, dosage, and duration, to enhance antimicrobial stewardship practices and combat antimicrobial resistance, ensuring effective and sustainable use of these essential medications^23,24^.

#### Evidence-based antimicrobial stewardship practice

Refers to healthcare professionals utilizing scientific evidence, clinical guidelines, and patient data to guide decisions on selecting, dosing, and timing antimicrobial treatment. Its objective is to enhance patient outcomes by reducing antimicrobial resistance and adverse effects, ensuring optimal treatment effectiveness^25–28^.

### Data extraction

The data were extracted from included studies using the data extraction tool prepared by MTB. The tool includes variables such as the name of the author, publication year, study design, data collection period, sample size, study area, and the point prevalence of antimicrobial use. The data extraction tool contains information on the indication for antibiotic use; prevalence of antibiotic use in different wards, classes of antibiotics used, types of antibiotics used, and AWaRe classification. BH extracted the data, and LWT and MTB cross-checked the extracted data for its validity and cleanness. Authors of papers were contacted to request missing or additional data.

### Data quality and risk of bias assessment

Eligible studies were critically appraised by two independent reviewers (MTB and BH). Full texts screening including the methodological quality assessment were examined using the JBI’s critical appraisal instrument for prevalence studies^29^. Studies that fulfill at least seven out of the nine domains of the JBI criteria questions were eligible for meta-analysis. The results of the critical appraisal were reported in narrative form and a table. A lower risk of bias (94%) observed after assessment (Appendix III). Studies with inadequate sample size, inappropriate sampling frame and poor data analysis were excluded. Articles were reviewed using titles, abstracts, and full text screening.

### Data analysis

Included studies were pooled in a statistical meta-analysis using STATA version 17.0. Effect sizes were expressed as a proportion with 95% confidence intervals around the summary estimate. Heterogeneity was assessed using the standard chi-square *I*^2^ test. A random-effects model was used. As pooled proportions from individual cross-sectional design point-prevalence studies are prone to variance instability and can violate the assumption of normality. Therefore, to address this, we did the double arcsine transformation method to stabilize variances, ensuring our meta-analysis results to be more reliable^30^. Sensitivity analyses were conducted to test decisions made regarding the included studies. Visual examination of funnel plot asymmetry (Appendix IV) and Egger’s regression tests were used to check for publication bias^31^. A Forest plot with 95% CI was computed to estimate the pooled point prevalence of evidence-based antimicrobial use among hospitalized patients in SSA.

### Protocol registration

The review protocol has been registered in PROSPERO with protocol registration number CRD42023404075.

### Ethical approval

Not applicable. Unlike primary studies, systematic reviews do not include the collection of deeply personal, sensitive, and confidential information from the study participants. Systematic reviews involve the use of publicly accessible data as evidence and are not required to seek an institutional ethics approval before commencement.

## Results

### Search

A total of 2260 articles were obtained from CINAHL, EMBASE, Google Scholar, PubMed, Scopus, and Web of Science databases. Following the removal of 605 duplicates, at the title/abstract screening phase (n = 2016) and during the full-article screening (n = 212) articles were excluded. Accordingly, 32 studies were eligible for quality assessment. Finally, 28 studies were included in this meta-analysis (Fig. 1).

### Study characteristics

The total sample size of this systematic review was 26, 272, ranging from 113 in Malawi^32^ to 4, 407 in South Africa^33^. Nine studies were reported from Nigeria^34–42^. Six articles were published from Ghana^43–48^. Four studies were reported from Kenya^49–52^. Equally two studies were reported from South Africa^33,53^ and Tanzania^54,55^. Bennin^56^, Botswana^57^, Ethiopia^58^, Malawi^32^, and Uganda^59^ reported only one study respectively (Table 1).

**Table 1:** Characteristics of included studies in the meta-analysis to assess point prevalence of evidence-based antibiotic use among hospitalized patients in sub-Saharan Africa.

S. no.	Author and year	Country	Study setting/no. of centers	Protocol used	Study period	Number of patients/Sample size	Point Prevalence of antibiotic use (%)	Prevalence of antibiotic use in different wards (%)	Indication for antibiotic use (%)	Classes of antibiotics used (%)	Types of antibiotic used (%)	AWaRe classification (%)
1	Usman (2020)	Nigeria	Hospital-wide/multicenter	ECDC protocol	April–May 2019	321	257/321 (80.1%)	Pediatric medical: 304	CI: 124	Nitroimidazole: 28.5	Metronidazole: 30.5	NA
								Neonatal: 298	HI: 52	Third-generation cephalosporin: 18.9	Ciprofloxacin: 17.1	
								Medical: 236	MP: 48	Fluoroquinolone: 13.6	Ceftriaxone: 16.8	
								Surgical: 251	SAP: 72	BLBLI: 10.5	Augmentin: 12.5	
								OBG: 234	Unknown: 24	Aminoglycoside: 8.5	Gentamicin: 11.8	
								Pediatric surgical: 290				
2	Aboderin et al. (2021)	Nigeria	Hospital-wide/multicenter	WHO protocol	10–27 June 2019	321	246/321 (76.6%)	Medical: 63	CI: 94	NA	Metronidazole: 25.2	Access: 46.3
								Surgical: 74	HI: 28		Cefuroxime: 18.4%	Watch: 53.5
								Pediatric: 34	SAP: 118		Ceftriaxone: 13.7	Reserve: 0.2
								Ortho: 30	MP: 36		Ciprofloxacin: 10.6	
								NNW/NICU: 44	Others: 44		Gentamicin: 10.5	
								Gynecology: 22				
								Postnatal: 38				
3	Afriyie et al. (2020)	Ghana	Hospital-wide/bicentric	Global PPS protocol	May-19	NA	GPH: 65%	Medical: 56.6–73.7	CI: 79.5–100	NA	NA	NA
							KMH: 82%	Surgical: 46.7–50.0	HI: 0–20.5			
								Pediatric medical: 77.8–100	SAP: 59.1–72. 2			
								Pediatric surgical: 100	MP: 27.8–40.9			
								NNW: 100				
4	Ahoyo et al. (2012)	Benin Republic	Hospital-wide/multicenter	HELICS protocol	10–26 October 2012	3130	2023/3130 (64.6%)	NA	NA	Beta-lactam: 86.9%	NA	NA
										Cephalosporin: 17.4%		
										Quinolone: 8.5%		
										Imidazole: 7.5		
										Aminoglycoside: 6.0%		
5	Amponsah et al. (2021)	Ghana	Hospital-wide/multicenter	WHO protocol	November–December 2019	190	115/190 (60.5%)	NA	CI: 36.5	Penicillin: 48.7%	Amoxicillin: 36.5	NA
									HI: 15.7	Cephalosporin: 23.5	Ciprofloxacin: 17.4	
									SAP: 26.1	Quinolone: 17.4	Ceftriaxone: 11.3	
									MP: 13.9	Lincosamide: 4.4	Cefuroxime: 9.6	
									Others: 7.8	Aminoglycoside: 2.6	Ampicillin: 7.8	
6	Bediako-Bowan et al. (2019)	Ghana	Surgical unit/multicenter	ECDC protocol	September–December 2016	540	382/540 (70.7%)	NA	CI: 174/382 (45.5%)	Nitroimidazole: 25.6	NA	NA
									HI: 50/382 (13.1%)	Second- and third-generation cephalosporin: 20.0		
									MP: 23/ (6.0%)	BLBLI: 16.7		
									SAP: 121 (31.7%)	Quinolone: 12.3		
									Unknown: 14	Lincosamide: 10.2		
7	Bunduki et al. (2021)	Malawi	Surgery department/single center	Adapted ECDC protocol	9-Jun-20	113	29/113 (27.6%)	NA	Prophylaxis: 10.3%	3^rd^ gen cephalosporin: 51.7%	Ceftriaxone: 51.7 Metronidazole: 44.8	NA
									Treatment: 48.3%	Metronidazole: 44.8	Amoxicillin: 24.1	
										Amoxicillin: 24.1	Doxycycline: 13.8	
										Doxycycline: 13.8	Ciprofloxacin: 13.8	
										Ciprofloxacin: 13.8		
7	Nsofor et al. (2016)	Nigeria	Hospital-wide/multicenter	ESAC protocol	NA	1585	886/1585 (55.9%)	NA	NA	NA	Chloramphenicol: 33.3	NA
											Tetracycline: 33.2	
											Ampicillin: 29.3	
											Amoxicillin: 28.9	
											Erythromycin: 26.4	
9	Fentie et al. (2022)	Ethiopia	Hospital-wide/multicenter	WHO PPS protocol	Jan-21	1820	1162	Surgical: 1208	CI: 615	NA	NA	NA
								Medical: 1065	HI: 733			
								OBG: 925	SAP: 333			
								NICU: 1385	MP: 131			
								Pediatric medical: 1396	Unknown: 55			
								ICU: 1565				
								Pediatric surgical: 1332				
								PICU: 1259				
10	Horumpende et al. (2020)	Tanzania	Hospital-wide/multicenter	ECDC protocol	November–December 2016	399	176	Medical: 140	CI: 168	Ceftriaxone: 28.5	Ceftriaxone: 28.5	NA
								Surgical: 160	HI: 40	Metronidazole: 23.9	Metronidazole: 23.9	
									SAP: 120	Penicillins: 26.9	Ampiclox:8.5 ampicillin: 7%	
									MP: 2	Aminoglycoside: 6.6	Gentamicin: 6.6	
									Unknown: 44	Cotrimoxazole: 3.9%		
11	Kamita et al. (2022)	Kenya	Hospital-wide/single center	Adapted global PPS protocol	Jul-21	308	191	ICU: 308	CI: 106	NA	NA	Access: 57
								Pediatric: 290	HI: 4			Watch: 42
								Medical: 213	SAP: 45			
								Gynecology: 202	MP: 38			
								Surgical: 197	Unknown: 111			
								Postnatal: 173	Others: 4			
								Neonatal: 140				
12	Fowotade et al. (2020)	Nigeria	Hospital-wide/single center	Global PPS protocol	Dec-17	451	426	NA ???	CI: 119	Cephalosporin: 30%	Ceftriaxone: 15.6%	NA
									HI: 53	Metronidazole: 18	Metronidazole: 14.6	
									SAP: 176	BLBLI: 16	Augmentin: 11.6	
									MP: 75	Aminoglycoside: 11	Ciprofloxacin: 9.1	
									Unknown: 7	Quinolones: 15	Gentamicin: 8.6%	
13	Kiggundu et al. (2022)	Uganda	Hospital-wide/multicenter	WHO PPS protocol	December 2020–April 2021	1077 patients	794	NA ???	CI: 448	NA	Ceftriaxone: 37%	Access: 47.2
									HI: 68		Metronidazole: 27%	Watch: 44.1
									SAP: 248		Gentamicin: 7%	Unclassified: 9.0
									MP: 313		Ampicillin: 6%	Reserve: 0.0
											Ampiclox: 6%	
14	Labi et al. (2018)	Ghana	Hospital-wide/single center	ESAC protocol	February–March 2016	677	348	OBG: 244	CI: 271	Penicillin: 24.9%	Metronidazole: 17.5	NA
								Pediatric surgical: 615	HI: 142	Nitroimidazole: 17.5%	Augmentin: 13.4%	
								Gynecology: 303	SAP: 227	Third-generation cephalosporin: 13.8	Ceftriaxone: 12.1%	
								Medical: 339	MP: 37	Second-generation cephalosporin: 10.0	Cefuroxime: 10.0%	
								Surgery: 385		Aminoglycoside: 8.8	Cloxacillin: 8.5%	
								Pediatric: 470				
15	Labi et al. (2021)	Ghana	Hospital-wide/multicenter	Global PPS	September–December 2019	2897	1562	Medical: 1486	SAP: 756	NA	Metronidazole: 20.6%	NA
								Surgical: 1449	MP: 232		Cefuroxime: 12.9% Ceftriaxone: 11.8%	
								IUC: 2587	Unknown: 397		Amoxicillin/clavulanic acid: 8.8%	
								Neo medical: 1828			Ciprofloxacin: 7.8%	
								NICU: 1538				
								Pediatric medical: 2121				
								Pediatric surgical: 1643				
								PICU: 1327				
16	Labi et al. (2018)	Ghana	Pediatric units/multicenter	Adapted ECDC protocol	September–December 2016	716	506	NA ???	CI: 437	Third-generation cephalosporin: 18.5%	NA	NA
									HI: 74	Aminoglycoside: 17.9%		
									Prophylaxis: 170	Second-generation cephalosporin: 12.4		
									Unknown: 34	Beta-lactam-resistant penicillin: 10.0		
										Nitroimidazole: 9.9		
17	Momanyi et al. (2019)	Kenya	Hospital-wide/single center	Global PPS	Apr-17	179	98	ICU: 179	CI: 97	Penicillin: 46.9	Ceftriaxone: 39.7%	NA
								Neonatal: 168	HI: 5	Cephalosporins: 44.7	Benzylpenicillin: 29.0%	
								Pediatric medical: 171	SAP: 47	Aminoglycosides: 26.3	Metronidazole: 25.1%	
								Medical: 110	MP: 27		Gentamicin: 22.3%	
								Surgical: 103			Flucloxacillin:11.2	
								OBG: 37				
18	Nnadozie et al. (2021)	Nigeria	Hospital-wide/single center	Global PPS	May-19	127	106	NA ???	CI: 83	NA	Ceftriaxone: 25.7	NA
									HI: 7		Tinidazole: 21.9	
									Prophylaxis: 37		Metronidazole: 14.6	
									Unknown: 0.3		Cefuroxime: 7.0	
											Levofloxacin: 5.6	
19	Oduyebo et al. (2017)	Nigeria	Hospital-wide/multicenter	NA	April–June 2015	828	577	ICU: 736	CI: 468	Third-generation cephalosporin: 21.4%	NA	NA
								Pediatric medical: 700	HI: 55	Metronidazole: 18.0		
								NICU: 636	SAP: 277	Quinolones: 14.1		
								Pediatric. surgical: 585	MP: 120			
								Surgical: 561	Unknown: 102			
								Medical: 524				
								Neonatal medical: 502				
								Hematology/oncology: 207				
20	Ogunleye et al. (2022)	Nigeria	Hospital-wide/bicentric	Adapted ECDC and global PPS protocol	Nov-19	491	398	NA ???	CI: 204	Cephalosporin: 43.5%	Ceftriaxone: 26.0%	NA
									HI: 28	Nitroimidazole: 28.8%	Metronidazole: 28.8%	
										Penicillins: 11.0%	Augmentin: 8.9%	
										Quinolones: 5.8%	Cefuroxime: 5.4%	
										Aminoglycoside: 4.4%	Levofloxacin: 3.5%	
21	Okoth et al. (2018)	Kenya	Hospital-wide/single center	Global PPS	5–12 June 2017	269	182	Postnatal: 249	CI: 75	Third-gen cephalosporin: 55%	NA	NA
								Neonatal: 224	HI: 35	Imidazole: 41.8		
								ICU: 179	SAP: 59	Broad spectrum penicillin: 41.8%		
								Medical: 173	MP: 78	Aminoglycoside: 7.1%		
								Gynecology: 173	Others: 16			
								Surgical: 167	Unknown: 5			
								Pediatrics: 158				
22	Omulo et al. (2022)	Kenya	Hospital-wide/multicenter	WHO protocol	September 2017 and March–April 2018	1071	489	ICU: 878	NA ???	NA	NA	NA
								Medical: 407				
								OBG: 514				
								Pediatric: 632				
								Surgical: 428				
23	Seni et al. (2020)	Tanzania	Hospital-wide/multicenter	WHO protocol	Dec-19	948	591	Medical: 454	CI: 377	NA	Ceftriaxone: 30.9%	Access: 97.9
								Surgical: 781	HI: 51		Metronidazole: 22.9%	Watch: 1.8
								Pediatric: 799	SAP: 273		Ampicillin–cloxacillin: 17.0%	Reserve: 0.3
								ICU: 611	MP: 216		Gentamicin: 11.0%	
											Ampicillin: 6.9%	
24	Skosana et al. (2021)	South Africa	Hospital-wide/multicenter	ECDC and global PPS	April–August 2018	4407	1479	NA ???	NA ???	NA	NA	Access: 54.6
												Watch: 30.2
												Reserve: 1.9
												Unclassified: 13.3
25	Skosana et al. (2021)	South Africa	Pediatric/multicenter	ECDC protocol	April–August 2018	1261	627	Pediatric medical: 942	Prophylaxis: 207	NA	Ampicillin: 16.4%	Access: 55.9
								Pediatric surgical: 121	Treatment: 1054		Gentamicin: 10.0%	Watch: 27.8
								PICU: 198			Amoxicillin/enzyme inhibitor: 9.6%	Reserve: 3.1
											Ceftriaxone: 7.4%	Unclassified: 13.2
											Amikacin: 6.3%	
26	Umeokonkwo et al. (2019)	Nigeria	Hospital-wide/single center	Global PPS protocol	October–November 2017	220	172	ICU: 220	CI: 100	Metronidazole: 33.9	NA	NA
								Adult surgical: 182	HI: 13	Third-generation cephalosporin: 37.5%		
								Pediatric medical: 182	SAP: 97	Second-generation cephalosporin: 7.7		
								Neonatal medical: 171	MP: 6			
								Pediatric surgical: 165	Unknown: 4			
								Adult medical: 156				
27	Manga et al. (2021)	Nigeria	Hospital-wide/single center	Global PPS protocol	Apr-19	326	235	Medical: 230	NA ???	Cephalosporins:29.2%	NA	NA
								Pediatric: 239		Penicillins: 22. 8%		
										Fluoroquinolones: 12.4		
										Aminoglycosides: 9.1		
										Macrolides: 3.4		
28	BD. A Paramadhas et al. (2019)	Botswana	All hospital sectors	Global and European PPS		711	502	PICU: 6, OBY: 199, AMW: 192, ASW: 164, PSW: 31, AICU: 17, PMW: 59, NICU: 43	CAI: 439, HAI: 60, HBCI: 3, NIC: 209	Metronidazole Parenteral: 252, Third generation ceftrioxone: 52, Cefotaxime: 398,		

ECDC, European Center for Diseases Prevention and Control; CAI, community acquired infection; HAI, hospital acquired infection; ICU, intensive care unit; PICU, pediatric intensive care unit; NICU, neonatal intensive care unit; PPS, point prevalence survey.

### Antibiotic use by wards among hospitalized patients in sub-Saharan Africa

The use of antibiotics from highest to lowest were surgical (5764), medical (5440), intensive care (4676), obstetrics and gynecology (2410), neonatal (830), oncology (207), and orthopedic (30) wards respectively (Table 2).

**Table 2:** Antibiotic use by wards among hospitalized patients in sub-Saharan Africa.

Authors and country	AU in medical wards n(%)	AU in Surgical ward n(%)	AU in Oby Gyn n(%)	AU in ICU Ward n(%)	AU in Neonatal ward n(%)	Hematology/Oncology n(%)	Orthopedics n(%)	Number of patients (N)
Usman et al. (2020)[Nigeria]	236 (73.5%)	251 (78.2%)	234 (72.9%)		298 (92.8%)			321
Fentie et al. (2022)[Ethiopia]	1065 (58.5%)	1208 (66.4%)	925 (50.8%)	1565 (85.9%)				1820
Horumpende et al. (2020)[Tanzania]	140 (35%)	160 (40.1%)						399
Kamita et al. (2022)[Kenya]	213 (69.2%)	197 (63.9%)	202 (65.6%)	308 (100%)	140 (45.5%)			308
Labi et al. (2018)[Ghana]	339 (59.6%)	385 (57.5%)	303 (45.3%)					669
Aboderin et al. (2021)[Nigeria]	63 (19.6%)	74 (23%)	22 (6.9%)				30 (9.4%)	321
Labi et al. (2021)[Ghana]	1486 (51.3%)	1449 (50%)						2897
Momanyi et al. (2019)[Kenya]	110 (61.5%)	103 (52.3%)	37 (18.8%)	179 (100%)	168 (85.3%)			179
Oduyebo et al. (2017)[Nigeria]	524 (63.3%)	561 (67.8%)		736 (88.9%)		207 (25%)		828
Okoth et al. (2018)[Kenya]	173 (64.3%)	167 (62.1%)	173 (64.3%)	179 (66.5%)	224 (83.3%)			269
Omulo et al. (2022)[Kenya]	407 (38%)	428 (39.9%)	514 (47.9%)	878 (81.9%)				1071
Seni et al. (2020)[Tanzania]	454 (47.9%)	781 (82.4%)		611 (64.5%)				948
Manga et al. (2021)[Nigeria]	230 (70.6%)							326

AU, antibiotic use.

### Most commonly used antibiotics among hospitalized patients in sub-Saharan Africa

Ceftriaxone^32–34,37,39–41,45–47,52,54,55,60,61^, metronidazole^32,34,37,39,40,42–44,46,47,52,54,55,59^, gentamicin^33,34,37,39,46,47,52,54,55,59^, ampicillin^33,38,46,54,55,60^, and cefuroxime^37,40,42,44–46^ were the most commonly used antibiotics (Table 3). Six studies equally reported ciprofloxacin^32,34,37,39,44,46^ and amoxicillin-clavulanate^33,34,39,42,61,62^. Only three studies reported ampicillin-cloxacillin combination^39,54,59^ and amoxicillin^32,38,46^ as antibiotics used in hospitals in SSA (Table 3).

**Table 3:** Most commonly used antibiotics among hospitalized patients in sub-Saharan Africa.

S. no.	Author and year	Country	Study setting/no. of centers	Classes of antibiotics used (%)	Types of antibiotic used (%)	AWaRe classification (%)
1	Usman (2020)	Nigeria	Hospital-wide/multicenter	Nitroimidazole: 28.5	Metronidazole: 30.5	NA
				Third-generation cephalosporin: 18.9	Ciprofloxacin: 17.1	
				Fluoroquinolone: 13.6	Ceftriaxone: 16.8	
				BLBLI: 10.5	Augmentin: 12.5	
				Aminoglycoside: 8.5	Gentamicin: 11.8	
2	Aboderin et al. (2021)	Nigeria	Hospital-wide/multicenter	NA	Metronidazole: 25.2	Access: 46.3
					Cefuroxime: 18.4%	Watch: 53.5
					Ceftriaxone: 13.7	Reserve: 0.2
					Ciprofloxacin: 10.6	
					Gentamicin: 10.5	
3	Ahoyo et al. (2012)	Benin Republic	Hospital-wide/multicenter	Beta-lactam: 86.9%	NA	NA
				Cephalosporin: 17.4%		
				Quinolone: 8.5%		
				Imidazole: 7.5		
				Aminoglycoside: 6.0%		
4	Amponsah et al. (2021)	Ghana	Hospital-wide/multicenter	Penicillin: 48.7%	Amoxicillin: 36.5	NA
				Cephalosporin: 23.5	Ciprofloxacin: 17.4	
				Quinolone: 17.4	Ceftriaxone: 11.3	
				Lincosamide: 4.4	Cefuroxime: 9.6	
				Aminoglycoside: 2.6	Ampicillin: 7.8	
5	Bediako-Bowan et al. (2019)	Ghana	Surgical unit/multicenter	Nitroimidazole: 25.6	NA	NA
				Second- and third-generation cephalosporin: 20.0		
				BLBLI: 16.7		
				Quinolone: 12.3		
				Lincosamide: 10.2		
6	Bunduki et al. (2021)	Malawi	Surgery department/single center	3^rd^ gen cephalosporin: 51.7%	Ceftriaxone: 51.7 Metronidazole: 44.8	NA
				Metronidazole: 44.8	Amoxicillin: 24.1	
				Amoxicillin: 24.1	Doxycycline: 13.8	
				Doxycycline: 13.8	Ciprofloxacin: 13.8	
				Ciprofloxacin: 13.8		
7	Nsofor et al. (2016)	Nigeria	Hospital-wide/multicenter	NA	Chloramphenicol: 33.3	NA
					Tetracycline: 33.2	
					Ampicillin: 29.3	
					Amoxicillin: 28.9	
					Erythromycin: 26.4	
8	Fentie et al. (2022)	Ethiopia	Hospital-wide/multicenter	NA	NA	NA
9	Horumpende et al. (2020)	Tanzania	Hospital-wide/multicenter	Ceftriaxone: 28.5	Ceftriaxone: 28.5	NA
				Metronidazole: 23.9	Metronidazole: 23.9	
				Penicillins: 26.9	Ampiclox:8.5 ampicillin: 7%	
				Aminoglycoside: 6.6	Gentamicin: 6.6	
				Cotrimoxazole: 3.9%		
10	Kamita et al. (2022)	Kenya	Hospital-wide/single center	NA	NA	Access: 57
						Watch: 42
11	Fowotade et al. (2020)	Nigeria	Hospital-wide/single center	Cephalosporin: 30%	Ceftriaxone: 15.6%	NA
				Metronidazole: 18	Metronidazole: 14.6	
				BLBLI: 16	Augmentin: 11.6	
				Aminoglycoside: 11	Ciprofloxacin: 9.1	
				Quinolones: 15	Gentamicin: 8.6%	
12	Kiggundu et al. (2022)	Uganda	Hospital-wide/multicenter	NA	Ceftriaxone: 37%	Access: 47.2
					Metronidazole: 27%	Watch: 44.1
					Gentamicin: 7%	Unclassified: 9.0
					Ampicillin: 6%	Reserve: 0.0
					Ampiclox: 6%	
13	Labi et al. (2018)	Ghana	Hospital-wide/single center	Penicillin: 24.9%	Metronidazole: 17.5	NA
				Nitroimidazole: 17.5%	Augmentin: 13.4%	
				Third-generation cephalosporin: 13.8	Ceftriaxone: 12.1%	
				Second-generation cephalosporin: 10.0	Cefuroxime: 10.0%	
				Aminoglycoside: 8.8	Cloxacillin: 8.5%	
14	Labi et al. (2021)	Ghana	Hospital-wide/multicenter	NA	Metronidazole: 20.6%	NA
					Cefuroxime: 12.9% Ceftriaxone: 11.8%	
					Amoxicillin/clavulanic acid: 8.8%	
					Ciprofloxacin: 7.8%	
15	Labi et al. (2018)	Ghana	Pediatric units/multicenter	Third-generation cephalosporin: 18.5%	NA	NA
				Aminoglycoside: 17.9%		
				Second-generation cephalosporin: 12.4		
				Beta-lactam-resistant penicillin: 10.0		
				Nitroimidazole: 9.9		
16	Momanyi et al. (2019)	Kenya	Hospital-wide/single center	Penicillin: 46.9	Ceftriaxone: 39.7%	NA
				Cephalosporins: 44.7	Benzylpenicillin: 29.0%	
				Aminoglycosides: 26.3	Metronidazole: 25.1%	
					Gentamicin: 22.3%	
					Flucloxacillin:11.2	
17	Nnadozie et al. (2021)	Nigeria	Hospital-wide/single center	NA	Ceftriaxone: 25.7	NA
					Tinidazole: 21.9	
					Metronidazole: 14.6	
					Cefuroxime: 7.0	
					Levofloxacin: 5.6	
18	Oduyebo et al. (2017)	Nigeria	Hospital-wide/multicenter	Third-generation cephalosporin: 21.4%	NA	NA
				Metronidazole: 18.0		
				Quinolones: 14.1		
19	Ogunleye et al. (2022)	Nigeria	Hospital-wide/bicentric	Cephalosporin: 43.5%	Ceftriaxone: 26.0%	NA
				Nitroimidazole: 28.8%	Metronidazole: 28.8%	
				Penicillins: 11.0%	Augmentin: 8.9%	
				Quinolones: 5.8%	Cefuroxime: 5.4%	
				Aminoglycoside: 4.4%	Levofloxacin: 3.5%	
20	Okoth et al. (2018)	Kenya	Hospital-wide/single center	Third-gen cephalosporin: 55%	NA	NA
				Imidazole: 41.8		
				Broad spectrum penicillin: 41.8%		
				Aminoglycoside: 7.1%		
21	Omulo et al. (2022)	Kenya	Hospital-wide/multicenter	NA	NA	NA
22	Seni et al. (2020)	Tanzania	Hospital-wide/multicenter	NA	Ceftriaxone: 30.9%	Access: 97.9
					Metronidazole: 22.9%	Watch: 1.8
					Ampicillin–cloxacillin: 17.0%	Reserve: 0.3
					Gentamicin: 11.0%	
					Ampicillin: 6.9%	
23	Skosana et al. (2021)	South Africa	Hospital-wide/multicenter	NA	NA	Access: 54.6
						Watch: 30.2
						Reserve: 1.9
						Unclassified: 13.3
24	Skosana et al. (2021)	South Africa	Pediatric/multicenter	NA	Ampicillin: 16.4%	Access: 55.9
					Gentamicin: 10.0%	Watch: 27.8
					Amoxicillin/enzyme inhibitor: 9.6%	Reserve: 3.1
					Ceftriaxone: 7.4%	Unclassified: 13.2
					Amikacin: 6.3%	
25	Umeokonkwo et al. (2019)	Nigeria	Hospital-wide/single center	Metronidazole: 33.9	NA	NA
				Third-generation cephalosporin: 37.5%		
				Second-generation cephalosporin: 7.7		
26	Manga et al. (2021)	Nigeria	Hospital-wide/single center	Cephalosporins:29.2%	NA	NA
				Penicillins: 22. 8%		
				Fluoroquinolones: 12.4		
				Aminoglycosides: 9.1		
				Macrolides: 3.4		
27	BD. A PARAMADHAS ET AL. (2019)	Botswana	all hospital sectors	Metronidazole Parenteral: 252, Third generation ceftrioxone: 52, Cefotaxime: 398,		
28	Daniel Ankrah (2021)	Ghana	Korle BuTeaching Hospital /multicenteric	Amoxicillin with beta-lactam inhibitor (17.5%), metronidazole (11.8%), ceftriaxone (11.5%)	Amoxicillin with beta-lactam inhibitor (17.5%), metronidazole (11.8%),ceftriaxone (11.5%)	

### WHO AWARE classification of antibiotics used by hospitalized patients in sub-Saharan Africa

Only five studies reported antibiotics used based on the WHO’s access, watch, and reserve (AWaRe) classification^33,37,49,53,59^ (Table 4). The most commonly used antibiotics were the access group and ranged between 46.3 and 97.9%^33,37,49,53,59^, followed by the watch and reserve group that accounted for 1.8–53.5%^33,37,49,53,59^, and 0.0–5.0%^33,37,49,53,59^ respectively (Table 4).

**Table 4:** WHO AWARE classification of antibiotics used by hospitalized patients in sub-Saharan Africa.

S. no.	Author and year	Country	Study setting/no. of centers	AWaRe classification (%)
1	Usman (2020)	Nigeria	Hospital-wide/multicenter	NA
2	Aboderin et al. (2021)	Nigeria	Hospital-wide/multicenter	Access: 46.3
				Watch: 53.5
				Reserve: 0.2
3	Afriyie et al. (2020)	Ghana	Hospital-wide/bicentric	NA
4	Ahoyo et al. (2012)	Benin Republic	Hospital-wide/multicenter	NA
5	Amponsah et al. (2021)	Ghana	Hospital-wide/multicenter	NA
6	Bediako-Bowan et al. (2019)	Ghana	Surgical unit/multicenter	NA
7	Bunduki et al. (2021)	Malawi	Surgery department/single center	NA
7	Nsofor et al. (2016)	Nigeria	Hospital-wide/multicenter	NA
9	Fentie et al. (2022)	Ethiopia	Hospital-wide/multicenter	NA
10	Horumpende et al. (2020)	Tanzania	Hospital-wide/multicenter	NA
11	Kamita et al. (2022)	Kenya	Hospital-wide/single center	Access: 57
				Watch: 42
12	Fowotade et al. (2020)	Nigeria	Hospital-wide/single center	NA
13	Kiggundu et al. (2022)	Uganda	Hospital-wide/multicenter	Access: 47.2
				Watch: 44.1
				Unclassified: 9.0
				Reserve: 0.0
14	Labi et al. (2018)	Ghana	Hospital-wide/single center	NA
15	Labi et al. (2021)	Ghana	Hospital-wide/multicenter	NA
16	Labi et al. (2018)	Ghana	Pediatric units/multicenter	NA
17	Momanyi et al. (2019)	Kenya	Hospital-wide/single center	NA
18	Nnadozie et al. (2021)	Nigeria	Hospital-wide/single center	NA
19	Oduyebo et al. (2017)	Nigeria	Hospital-wide/multicenter	NA
20	Ogunleye et al. (2022)	Nigeria	Hospital-wide/bicentric	NA
21	Okoth et al. (2018)	Kenya	Hospital-wide/single center	NA
22	Omulo et al. (2022)	Kenya	Hospital-wide/multicenter	NA
23	Seni et al. (2020)	Tanzania	Hospital-wide/multicenter	Access: 97.9
				Watch: 1.8
				Reserve: 0.3
24	Skosana et al. (2021)	South Africa	Hospital-wide/multicenter	Access: 54.6
				Watch: 30.2
				Reserve: 1.9
				Unclassified: 13.3
25	Skosana et al. (2021)	South Africa	Pediatric/multicenter	Access: 55.9
				Watch: 27.8
				Reserve: 3.1
				Unclassified: 13.2
26	Umeokonkwo et al. (2019)	Nigeria	Hospital-wide/single center	NA
27	Manga et al. (2021)	Nigeria	Hospital-wide/single center	NA

NA, not applicable.

### Indications for antibiotic prescription among hospitalized patients in SSA

Community-acquired infection ranged from 27.7 to 61%, surgical antibiotic prophylaxis ranged from 14.6 to 45.3%, hospital-acquired infections ranged from 1.2 to 40.3%, and, medical prophylaxis ranged from 0.5 to 29.1% were the most common clinical indications (Table 5). Antibiotic prescription for 938 inpatients were done for unknown clinical indications (Table 5).

**Table 5:** Clinical indications for which antibiotics were prescribed for hospitalized patients in sub-Saharan Africa.

Author and country	Community acquired infectionI n(%)	Hospital acquired infection n(%)	Medical prophylaxis n(%)	Surgical prophylaxis n(%)	Unkown n(%)	Number of patients (N)
Usman et al. (2020)[Nigeria]	124 (38.7%)	52 (16.3%)	48 (14.9)	72 (22.5%)	24 (7.6%)	321
Umeokonkwo et al. (2019)[Nigeria]	100 (45.5%)	13 (6%)	6(2.9%)	97 (44%)	4 (1.6%)	220
Aboderin et al. (2021)[Nigeria]	94 (29.2%)	28 (8.8%)	36 (11.2%)	118 (36.9%)	44 (13.8%)	321
Fowotade et al. (2020)[Nigeria]	119 (27.7%)	53 (12.3%)	75 (17.4%)	176 (40.9%)	7 (1.63%)	451
Nnadozie et al. (2021)[Nigeria]	83 (65%)	7 (5.3%)	37 (29.1%)		4(0.3%)	127
Oduyebo et al. (2017)[Nigeria]	468 (45.79%)	55 (5.38%)	120 (11.7)	277 (27.1%)	102 (9.9%)	828
Ogunleye et al. (2022)[Nigeria]	204 (41.5%)	28 (5.7%)				491
Labi et al. (2018)[Ghana]	271 (40.1%)	421 (21.0%)	37 (5.4%)	227 (33.6%)		677
Labi et al. (2021)[Ghana]			232 (8.0%)	756 (26.1%)	397 (13.7%)	2897
Labi et al. (2018)[Ghana]	437 (61.0%)	74 (10.3%)	170 (23.7%)		34 (4.8%)	716
Amponsah et al. (2021)[Ghana]	69 (36.5%)	30 (15.7%)	26 (13.9%)	50 (26.1)	15 (7.8%)	190
Bediako-B et al. (2019)[Ghana]	174 (45.5%)	50 (13.1%)	23 (6.0%)	121 (31.7%)	14 (3.7%)	540
Daniel A et al. (2021) [Ghana]	182 (18.4%)	110 (11.1%)			113 (11.4%)	988
Kamita et al. (2022)[Kenya]	106 (34.5%)	4 (1.2%)	38 (12.3)	45 (14.6%)	115 (36.3%)	308
Okoth et al. (2018)[Kenya]	75 (28%)	35 (13%)	78 (29%)	59 (22%)	21 (8%)	269
Horumpende et al. (2020)[Tanzania]	168 (42.0%)	40 (10%)	2 (0.5%)	120 (30%)	44 (11%)	399
Seni et al. (2020)[Tanzania]	377 (39.8%)	51 (5.4%)	216 (22.8%)	273 (28.8%)		948
Bunduki et al. (2021)[Malawi]			12 (10.3%)	55 (48.3%)		113
Fentie et al. (2022)[Ethiopia]	615 (33.8%)	733 (40.3%)	131 (7.2%)	333 (18.3%)	55 (0.3%)	1820
Kiggundu et al. (2022)[Uganda]	448 (41.6%)	68 (6.3%)	313 (29.1%)	248 (23.0%)		1077
Momanyi et al. (2019)[Kenya]	97 (54.2%)	5 (2.8%)	27 (15.1%)	47 (26.3%)		179
BDA Paramadhas et al. (2019)[Botswana]	439 (61.7%)	60 (8.4%)	3 (0.4%)	209 (29.4%)		711

### Pooled point prevalence of evidence-based use of antibiotics in SSA

**Figure 2:**
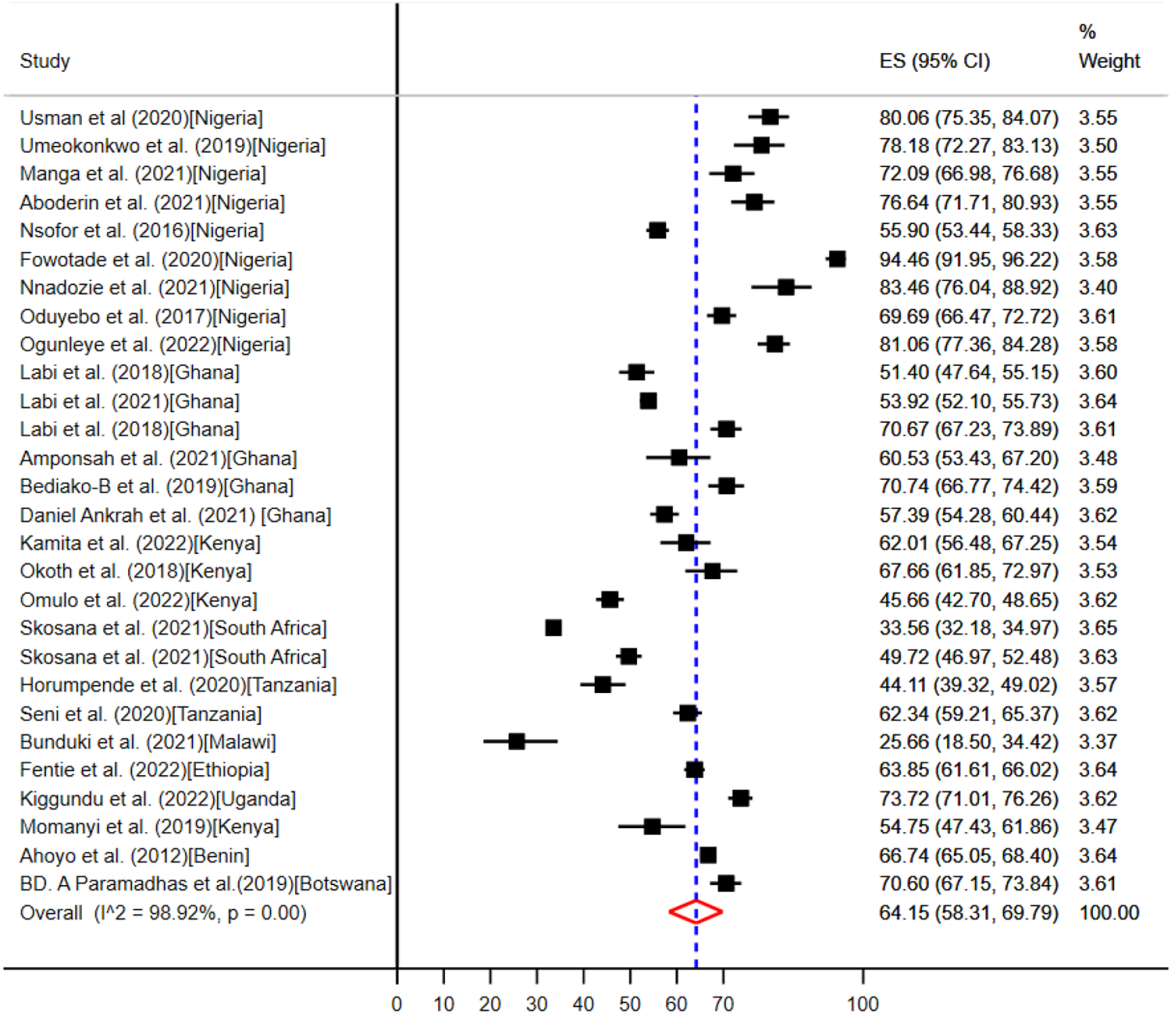
The pooled point prevalence of evidence-based use of antibiotics among hospitalized patients in sub-Saharan Africa.

The pooled point prevalence of evidence-based use of antimicrobials were 64.15% (95%CI: 58.31–69.79%) (Fig. 2).

## The pooled prevalence of evidence-based antibiotic use in different wards in hospitals of SSA

**Figure 3:**
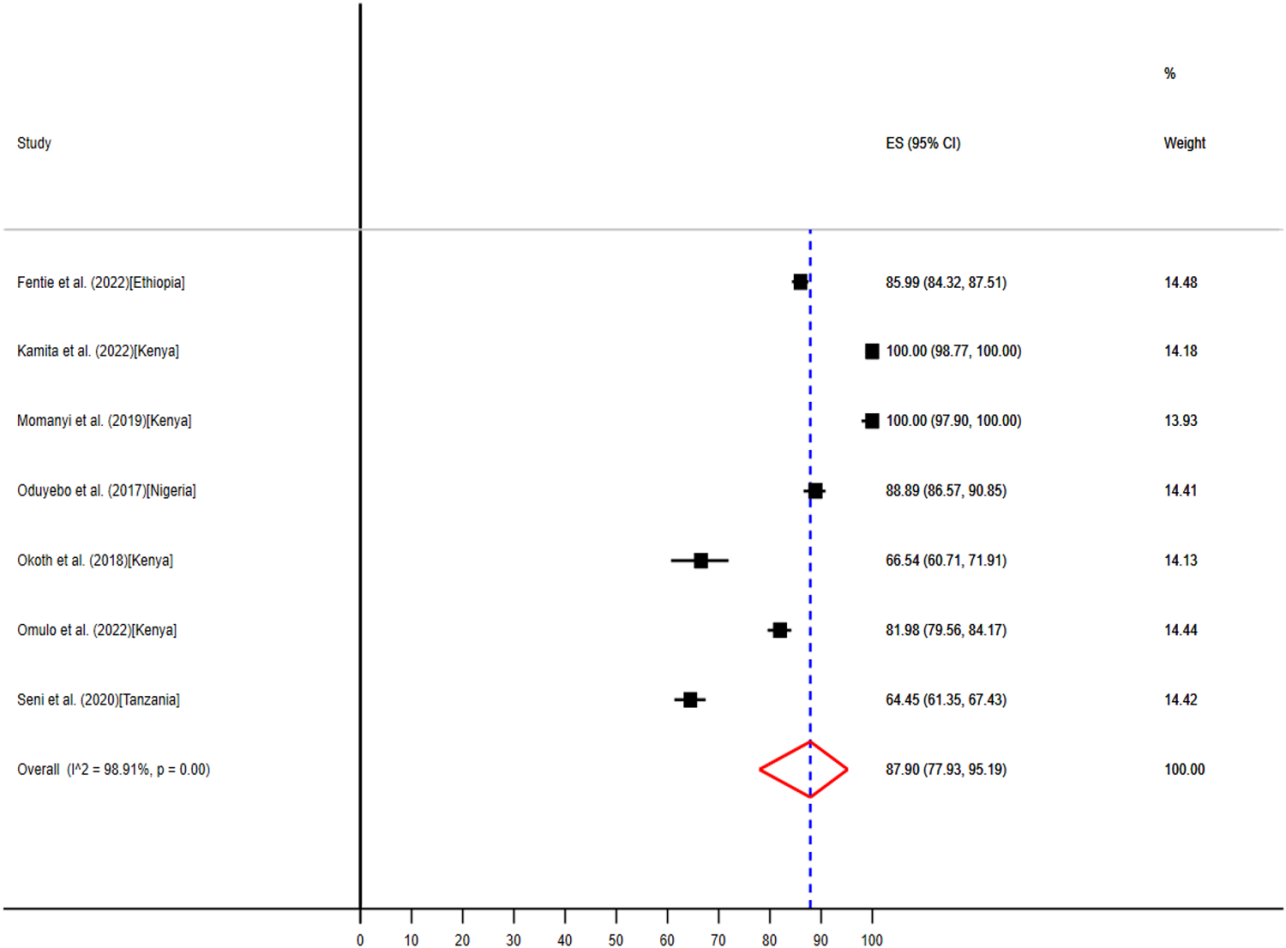
The pooled point prevalence of evidence-based use of antibiotics in intensive care units in hospitals of sub-Saharan Africa.

Only seven studies from four countries reported the use of antibiotics in intensive care units^41,49–52,55,58^, ranging from 179 (66.5%) to 1565 (85.9%) (Table 3). The pooled point prevalence of antibiotics use in ICU were 87.90% (95% CI: 77.93–95.19%) (Fig. 3).

**Figure 4:**
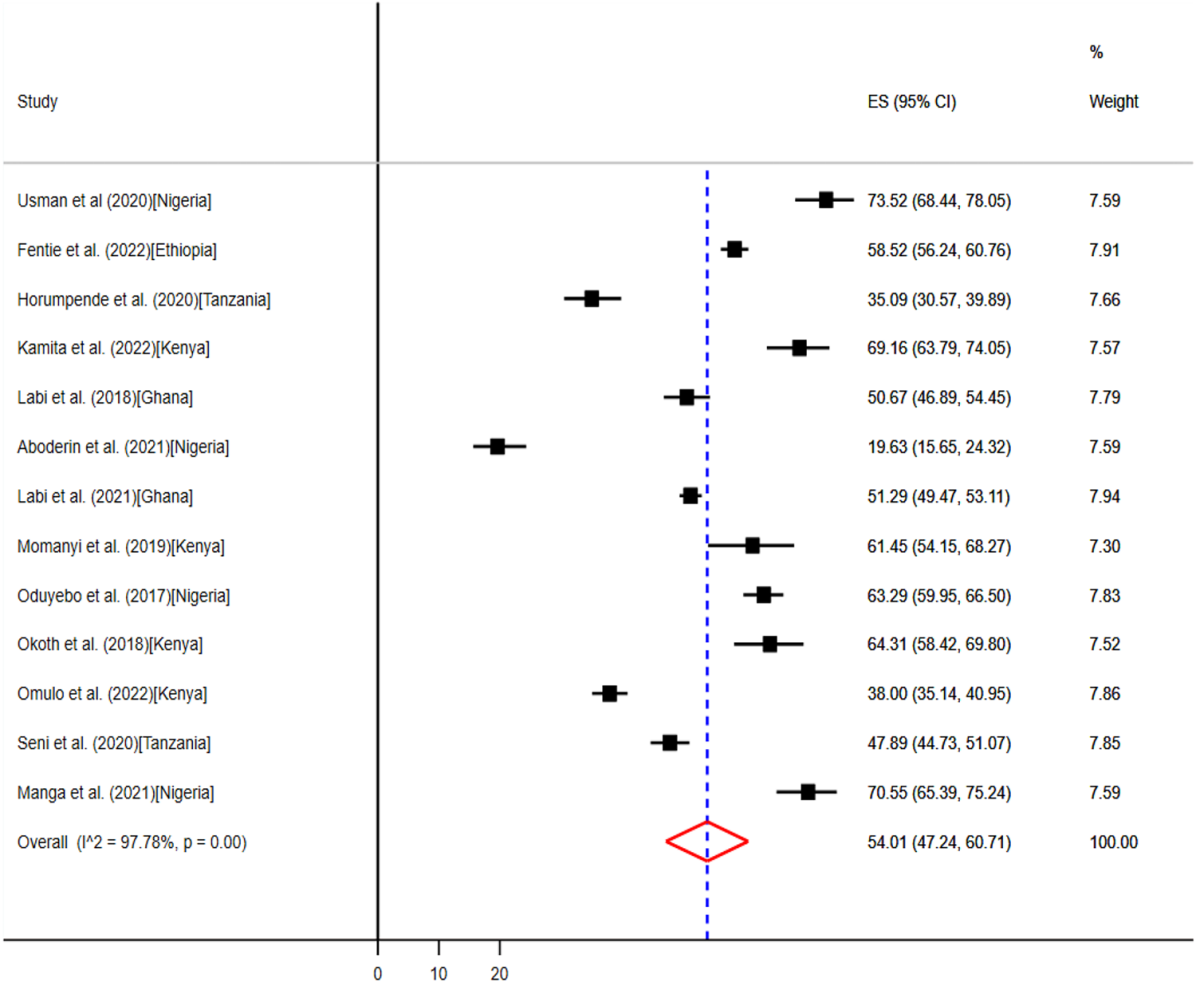
The pooled point prevalence of evidence-based use of antibiotics in medical wards in hospitals of sub-Saharan Africa.

The uptake of antimicrobials in medical wards ranged from 63 (19.6%) to 236 (73.5%) as reported by thirteen studies^34,36,37,41,43,49–52,54,55,58,61^ from five countries (Table 3). The pooled prevalence of use of antibiotics in medical wards were 54.01% (95% CI: 47.24–60.71%) (Fig. 4).

**Figure 5:**
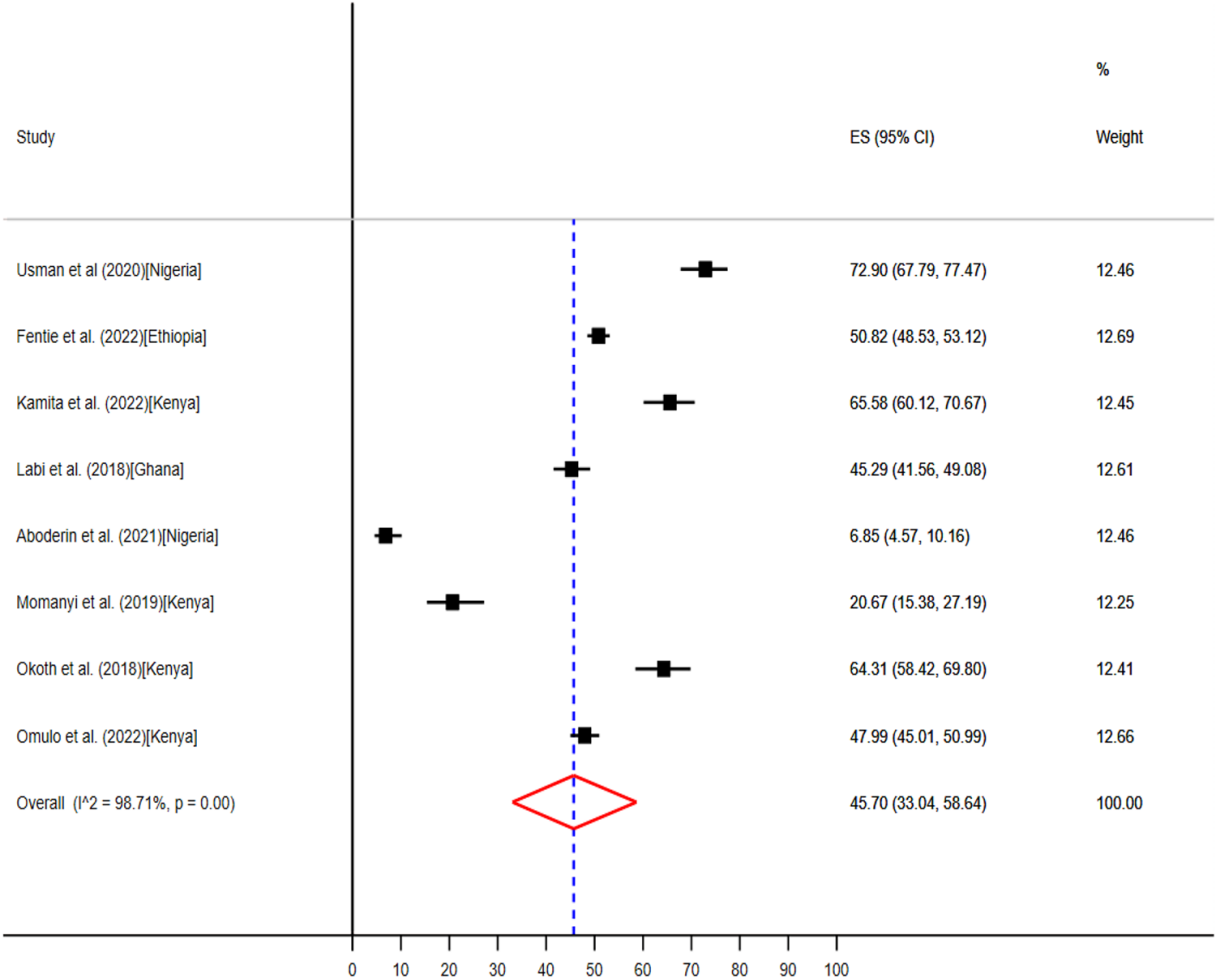
The pooled point prevalence of evidence-based use of antibiotics in obstetrics and gynecology wards in hospitals of sub-Saharan Africa.

Antibiotic use in obstetrics and gynecology wards ranges from 22 (6.9%) to 234 (72.9%)The pooled prevalence of antibiotics use in obstetrics and gynecology wards obtained from data extracted from eight studies published from Ethiopia^58^, Ghana^45^, Kenya^49–52^, and Nigeria^34,37^ (Table 3), were 45.70% (95% CI: 33.04–58.64) (Fig. 5).

**Figure 6:**
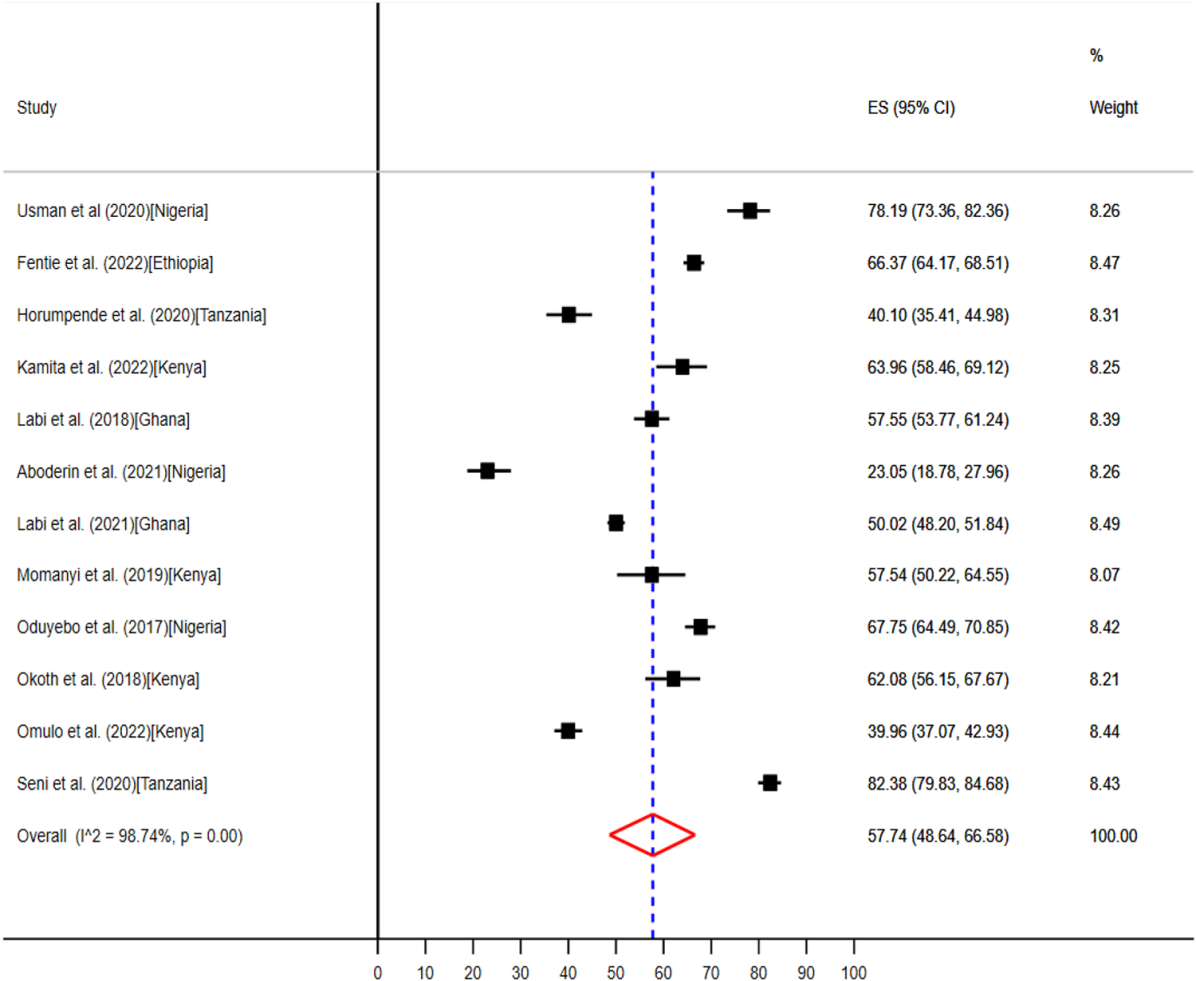
The pooled point prevalence of evidence-based use of antibiotics in surgical wards in hospitals of sub-Saharan Africa.

Five counties from hospitals in sub-Saharan Africa, including Ethiopia^58^, Ghana^61^, Kenya^49–52^, Nigeria^34,37,41^, and Tanzania^54,55^, produced twelve articles that revealed the antimicrobials uptake in surgical wards with the lowest 74 (23%) to the highest 781 (82.4%) (Table 3). The pooled prevalence of antibiotics use in surgical wards were 57.74% (95% CI: 48.64–66.58) (Fig. 6).

### The pooled prevalence of clinical indications for evidence-based antibiotic use in SSA

**Figure 7:**
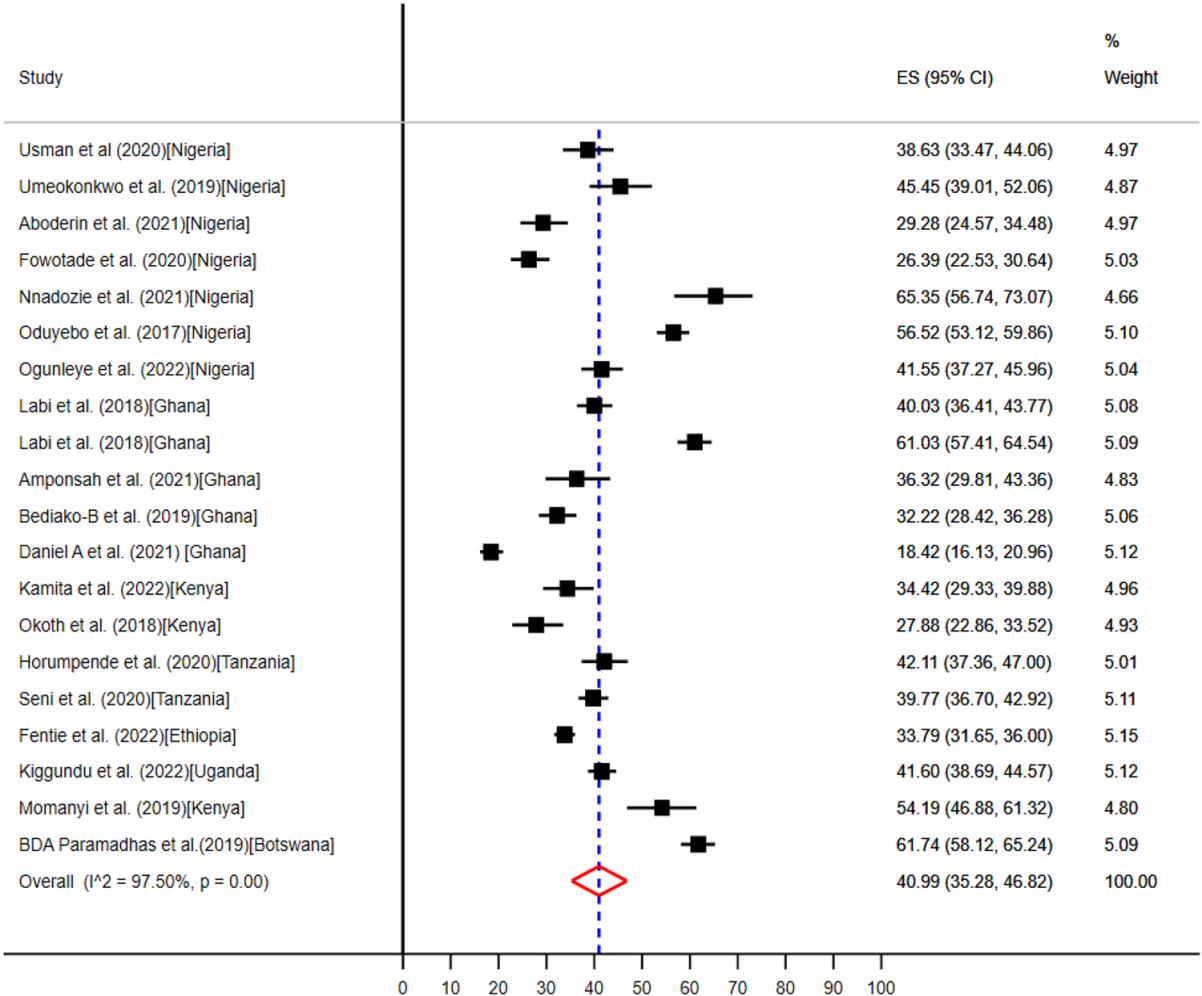
The pooled prevalence of evidence-based use of antibiotics for community acquired infections in hospitals of sub-Saharan Africa.

**Figure 8:**
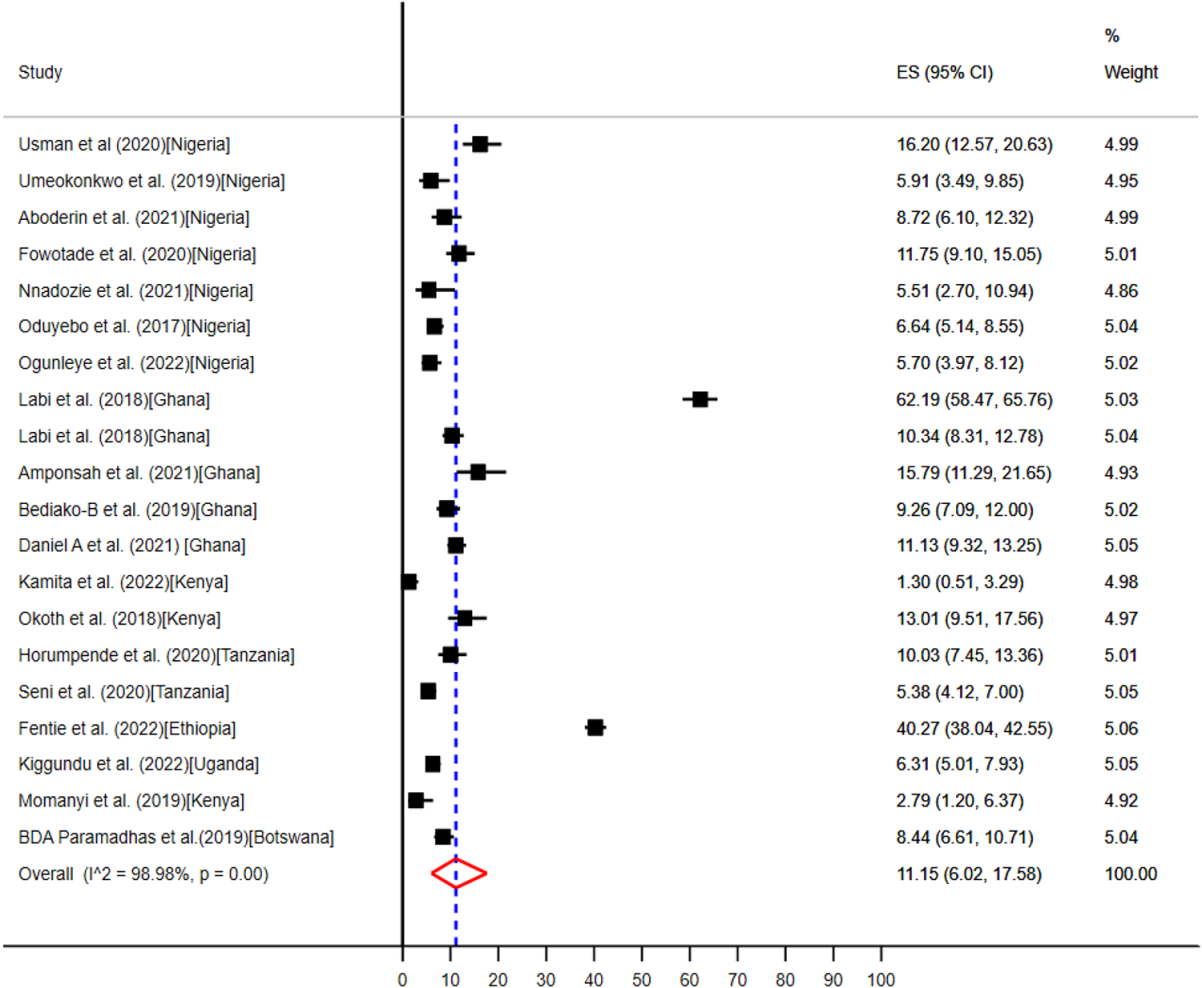
The pooled prevalence of evidence-based use of antibiotics for hospital acquired infections in hospitals of sub-Saharan Africa.

Twenty studies from seven countries in SSA such as, Botswana^57^, Ethiopia^58^, Nigeria^35,37,39–42,63^, Ghana^43,46–48,61^, Kenya^49,50,52^, Tanzania^54,55^, and Uganda^59^, reported that community- and hospital acquired infections were the most common clinical indications for antibiotics use (Table 5). The pooled prevalence of community- and hospital acquired infections for point of care antibiotics use were 40.99% (95% CI: 35.28–46.82%) (Fig. 7) and 11.15% (95% CI: 6.02–17.56%) (Fig. 8) respectively.

**Figure 9:**
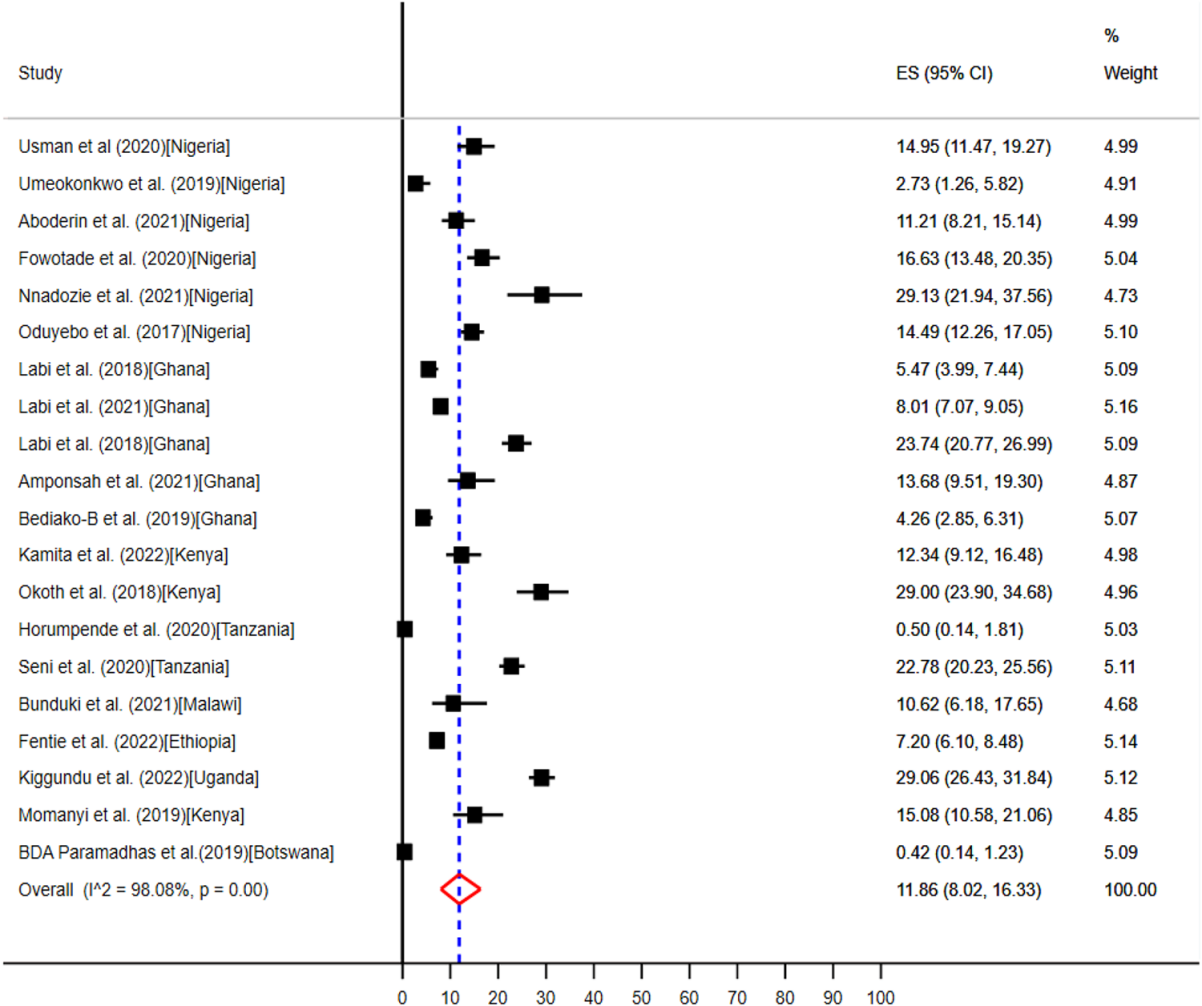
The pooled prevalence of evidence-based use of antibiotics for medical prophylaxis in hospitals of sub-Saharan Africa.

**Figure 10:**
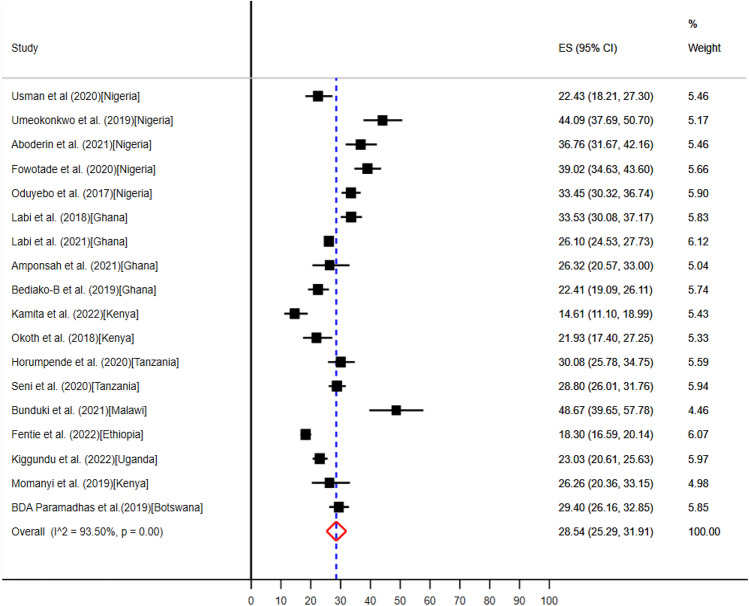
The pooled prevalence of evidence-based use of antibiotics for surgical prophylaxis in hospitals of sub-Saharan Africa.

Seven countries including Botswana^57^, Ethiopia^58^, Nigeria^34,35,37,39–41^, Ghana^45,47,61,64,65^, Kenya^49,50,52^, Tanzania^54,66^, Malawi^32^, and Uganda^59^ conducted eighteen studies which reported medical and surgical prophylaxis were the second most common clinical indications for evidence-based uptake of antimicrobials (Table 5). The pooled prevalence of medical—and surgical prophylaxis for antibiotics use were 11.86% (95% CI: 8.02–16.33%) (Fig. 9) and 28.54% (95% CI: 25.29–31.91%) (Fig. 10) respectively.

**Figure 11:**
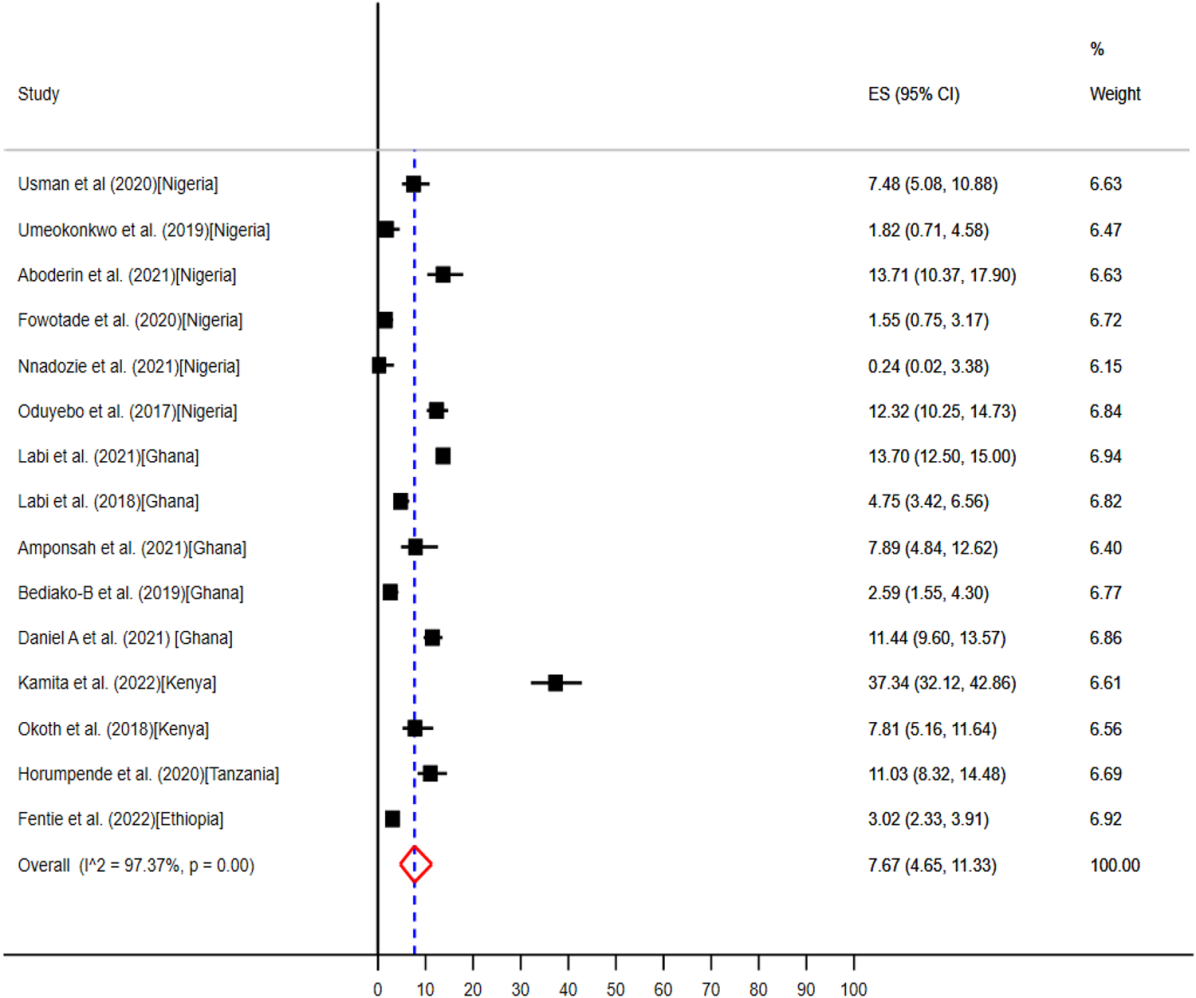
The pooled prevalence of evidence-based use of antibiotics for unknown clinical indications in hospitals of sub-Saharan Africa.

The pooled prevalence of the use of antibiotics at point of care for unknown clinical indications reported from 15 articles conducted in five countries Ethiopia^58^, Ghana^46–48,62,64^, Kenya^49,50^, Nigeria^34,35,37,39–41^, and Tanzania^54^ (Table 5) were 7.67% (95% CI: 4.55–11.33%) (Fig. 11).

Visual funnel plots asymmetry examination and Egger’s regression tests revealed that there was no publication bias^67^.

## Discussion

This systematic review and meta-analysis aimed to determine the pooled point prevalence of evidence-based antimicrobial use among hospitalized patients in sub-Saharan Africa. A total of 26, 272 patients admitted to twenty-eight hospitals of ten countries in SSA were included. The pooled point prevalence of antimicrobial use at point of care was 64%. The finding of this study is higher than the antibiotic use in hospitals of Middle East (28.3%)^68^ and Europe (30.5%)^69^. This could be attributed to misuse and overuse of antibiotics^70,71^, poor infection and disease prevention and control^72^, and, water, sanitation and hygiene practice in health-care facilities^73^, and poor surveillance of antimicrobial resistance in SSA^74,75^. The pooled point prevalence of antibiotic use in intensive care unit of hospitals in SSA were 89%. This finding is higher than a point prevalence of use of antimicrobials in ICUs in the United States 62.2% ^76^ and Poland 59.6%^77^.

The uses of antimicrobials at point of care in surgical and medical wards were 58% and 54% in SSA. The overuse or inappropriate use of antimicrobials at the point of care in medical and surgical wards can lead to antibiotic resistance^8^, which can make infections harder to treat. Moreover, unnecessary antimicrobial use can disrupt the balance of the microbiome, leading to complications like *Clostridium difficile* infections^78^. The pooled estimate of antibiotics used by inpatients admitted to obstetrics and gynecology wards of the hospitals in SSA were 46%. The finding of this study was higher than the antibiotic consumption in obstetrics and gynecology departments of Peruvian hospital 31%^79^. Higher antibiotic use in obstetrics and gynecology wards in SSA can be attributed to factors such as a higher prevalence of surgical procedures^80^, which often require prophylactic antibiotics to prevent post-operative infections^81^. Additionally, cases of infections related to childbirth, such as postpartum infections or complications following gynecological procedures, may necessitate antibiotic treatment in SSA^82,83^.

The pooled prevalence of community and hospital acquired infections in SSA were 41% and 11.15% respectively. The pooled estimate of this review was higher than a study in East Africa that reported 34% CAI^84^. This could be due to non-standardized antibiotic use in SSA. Our review result revealed that HAI in SSA were lower than the finding from LMICs 17.9%^85^.

The misuse of antibiotics in both community and hospital-acquired infections has far-reaching consequences^86^. In the community, inappropriate antibiotic use contributes to the development of antibiotic-resistant bacteria, rendering infections harder to treat and increasing healthcare costs^87,88^. Patients may experience treatment failures, longer hospital stays, and increased mortality rates^89^. Moreover, the continued misuse of antibiotics fuels the global crisis of antibiotic resistance, jeopardizing the effectiveness of these essential drugs for future generations^90,91^. In hospital settings, similar consequences are exacerbated by the potential for widespread outbreaks of antibiotic-resistant infections among vulnerable patients^92^. The resulting challenges in managing infections can strain healthcare systems, diminish the success of medical interventions, and underscore the critical need for stringent antibiotic stewardship practices to preserve the efficacy of antibiotics.

The pooled prevalence of the most common clinical indications for antibiotic use in hospitals of SSA were community acquired infection (40.99%), surgical prophylaxis (28.54%), medical prophylaxis (11.86%), and hospital acquired infection (11.15%).

This study revealed that the pooled prevalence of HAI (11.15%) is lower than the global estimate (14%)^93^. This could be attributed to inadequate infection control measures^94^, limited resources^95^, overcrowding^96^, and a higher burden of infectious diseases^97^. Poor sanitation and healthcare infrastructure can contribute to the increased risk of infections within healthcare facilities in SSA^98^.

According to this study, the pooled estimate of surgical prophylaxis is higher than Europe (16.8%)^99^ and the global surgical antibiotic prophylaxis at point of care (22.8%)^17^. The surgical prophylaxis in SSA is lower than a study reported in Myanmar (34.3%)^100^. Higher surgical antibiotic prophylaxis may be attributed to surgeon’s overuse of antibiotics to mitigate infection risks in environments with higher prevalence of surgical site infections and limited access to post-operative care in SSA^101–103^. Surgeons may also lack awareness of appropriate guidelines, and patients may expect antibiotics due to a perception of their effectiveness^103^.

The pooled point prevalence of medical prophylaxis in this study is lower than European region (24.9%)^69^ and Indonesia (47.1%)^104^. A lower point prevalence of medical prophylaxis in SSA suggests limited access and utilization of preventative medical interventions^105^. This may be indicative of healthcare system challenges, resource constraints, or insufficient awareness and education^106,107^. It can result in a higher disease burden, increased healthcare costs, and potentially poorer clinical and public health outcomes for the population^10,108^.

This review indicated that the pooled prevalence of community acquired infection is higher than a study conducted in the Middle East (16.8%)^68^. Community acquired infection in SSA according to this study were lower than Northern Ireland (66.2%)^109^. Higher prevalence of CAI could be due to lack of essential medical supplies, suboptimal sterilization procedures, and inadequate training in infection control^110,111^. High patient-to-nurse ratios and frequent patient turnover can further hinder the implementation of rigorous infection prevention measures, increasing the risk of infections spreading within healthcare settings^112,113^.

Antibiotic use for unknown clinical indications in SSA hospitals may occur due to inadequate training on antibiotic stewardship and a lack of access to timely microbiological testing^3,114^. Clinicians may resort to broad-spectrum antibiotics as a precautionary measure in the absence of specific diagnostic information, contributing to antibiotic misuse and resistance^114^.

## Conclusion

The pooled point prevalence of antimicrobial use among hospitalized patients were higher in SSA. Higher use of antibiotics in intensive care unit, surgical, medical, and obstetrics and gynecology wards of hospital in SSA were recorded. Community acquired infection, surgical and medica prophylaxis, and hospital acquired infection were clinical indications reported to have the highest to lowest pooled point prevalence of antibiotics used. Health systems in SSA must design innovative interventions to optimize clinicians adhere to evidence-based prescribing guidelines and improve antimicrobial stewardship.

### Implications for evidence-informed policy and clinical practice

A higher pooled point prevalence of antimicrobial use in sub-Saharan Africa implies a need for immediate policy and clinical practice interventions. Policymakers should prioritize allocation of scarce resources for antimicrobial stewardship programs and infection control measures. Innovative intervention must be in place to optimize clinicians adhere to evidence-based prescribing guidelines to combat antimicrobial resistance, reduce adverse effects, and improve patient outcomes.

Health systems in sub-Saharan Africa must emphasize the importance of leveraging clinical decision support digital health interventions to augment evidence-based antimicrobial stewardship. This evidence synthesis informs the policy decision makers to encourage the implementation of such tools to guide clinicians in evidence-based antimicrobial prescribing, reducing inappropriate use, combating resistance, and improving patient care in the context of resource constrained health system. Clinicians can benefit from real-time patient information, aiding in evidence-based prescribing and infection control efforts, significantly improving patient care. Collaboration between policymakers, clinicians, and healthcare facilities is crucial to mitigate the impact of these issues on public health.

### Supplementary Information

Supplementary Information.

## Data Availability

The datasets are available from the corresponding author on reasonable request.

## Abbreviations

AMR: Antimicrobial resistance
AHRI: The Armauer Hansen Research Institute
DDD: Defined daily dose
JBI: The Joanna Briggs Institute
LMICs: Low- and Middle-Income Countries
PRISMA: Preferred reporting items for systematic reviews and meta-analyses
PROSPERO: International prospective registry of systematic reviews
SDG: Sustainable development goal
SSA: Sub-Saharan Africa
WHO: The World Health Organization
