# Supplementary material for: Point prevalence of evidence-based antimicrobial use among hospitalized patients in sub-Saharan Africa: a systematic review and meta-analysis
Source: Sci Rep. 2024 Jun 2;14:12652. doi: 10.1038/s41598-024-62651-6 (PMC11144712; doi:10.1038/s41598-024-62651-6)

**Appendix I: Search strategy**

| **Databases** | **Search strategies (queries)** | **Result** |
| --- | --- | --- |
| PubMed | ((((((((((((point-prevalence[MeSH Terms]) AND (antibiotic use[MeSH Terms])) OR (antimicrobial use[MeSH Terms])) OR (rational antibiotic use[MeSH Terms])) OR (rational antimicrobial use[MeSH Terms])) OR (antimicrobial stewardship[MeSH Terms]) ) OR (point-prevalence[Text Word])) OR (antibiotic use[Text Word])) OR (antimicrobial use[Text Word])) OR (rational antibiotic use[Text Word])) OR (rational antimicrobial use[Text Word])) OR (antimicrobial stewardship[Text Word])) AND ((Sub Saharan Africa[Text Word]) OR (Angola[Text Word]) OR (Benin[Text Word]) OR (Botswana[Text Word]) OR (Burkina Faso[Text Word]) OR (Burundi[Text Word]) OR (Cabo Verde[Text Word]) OR (Cameroon[Text Word]) OR (Central African Republic[Text Word]) OR (Chad[Text Word]) OR (Comoros[Text Word]) OR (Congo[Text Word]) OR (Democratic Republic Congo[Text Word]) OR (Cote d’Ivoire[Text Word]) OR (Ivory Coast[Text Word]) OR (Djibouti[Text Word]) OR (Equatorial Guinea[Text Word]) OR (Eritrea[Text Word]) OR (Ethiopia[Text Word]) OR (Gabon[Text Word]) OR (Gambia[Text Word]) OR (Ghana[Text Word]) OR (Guinea[Text Word]) OR (Guinea-Bissau[Text Word]) OR (Kenya[Text Word]) OR (Lesotho[Text Word]) OR (Liberia[Text Word]) OR (Madagascar[Text Word]) OR (Malawi[Text Word]) OR (Mali[Text Word]) OR (Mauritania[Text Word]) OR (Mauritius[Text Word]) OR (Mozambique[Text Word]) OR (Namibia[Text Word]) OR (Niger[Text Word]) OR (Nigeria[Text Word]) OR (Reunion[Text Word]) OR (Rwanda[Text Word]) OR (Sao Tome[Text Word] AND Principe[Text Word]) OR (Senegal[Text Word]) OR (Seychelles[Text Word]) OR (Sierra Leone[Text Word]) OR (Somalia[Text Word]) OR (South Africa[Text Word]) OR (Sudan[Text Word]) OR (Swaziland[Text Word]) OR (Tanzania[Text Word]) OR (Togo[Text Word]) OR (Uganda[Text Word]) OR (Zambia[Text Word]) OR (Zimbabwe[Text Word]) OR (East Africa[Text Word]) OR (Middle Africa[Text Word]) OR (Southern Africa[Text Word]) OR (West Africa[Text Word]) OR (Central Africa[Text Word]) OR (Western Sahara[Text Word])) *Filters: from 2013/1/1 - 2023/12/31* | **1, 119** |
| Embase | 'point prevalence'/exp OR 'point prevalence' OR 'point prevalence survey'/exp OR 'point prevalence survey' AND 'antibiotic use'/exp OR 'antibiotic use' OR 'antimicrobial use'/exp OR 'antimicrobial use' OR 'antimicrobial stewardship'/exp OR 'antimicrobial stewardship' OR 'antimicrobial stewardship program'/exp OR 'antimicrobial stewardship program' AND 'angola'/exp OR 'benin'/exp OR 'botswana'/exp OR 'burkina faso'/exp OR 'burundi'/exp OR 'cape verde'/exp OR 'cameroon'/exp OR 'central african republic'/exp OR 'chad'/exp OR 'comoros'/exp OR 'democratic republic congo'/exp OR 'congo'/exp OR 'cote d`ivoire'/exp OR 'equatorial guinea'/exp OR 'eritrea'/exp OR 'eswatini'/exp OR 'ethiopia'/exp OR 'gabon'/exp OR 'gambia'/exp OR 'ghana'/exp OR 'guinea'/exp OR 'guinea-bissau'/exp OR 'kenya'/exp OR 'lesotho'/exp OR 'liberia'/exp OR 'madagascar'/exp OR 'malawi'/exp OR 'mali'/exp OR 'mauritania'/exp OR 'mauritius'/exp OR 'mozambique'/exp OR 'namibia'/exp OR 'niger'/exp OR 'nigeria'/exp OR 'rwanda'/exp OR 'sao tome and principe'/exp OR 'senegal'/exp OR 'seychelles'/exp OR 'sierra leone'/exp OR 'somalia'/exp OR 'south africa'/exp OR 'south sudan'/exp OR 'sudan'/exp OR 'tanzania'/exp OR 'togo'/exp OR 'uganda'/exp OR 'zambia'/exp OR 'zimbabwe'/exp AND [2013-2023]/py | **75** |
| CINAHL | ''point prevalence*'' OR ''point prevalence survey*'' AND TX ''antibiotic use*'' OR TX ''antimicrobial use*'' OR TX "antimicrobial stewardship*" OR TX "antimicrobial stewardship program*" OR TX "rational antimicrobial use*" AND TX ( Angola OR Benin OR Botswana OR Burkina AND faso OR Burundi OR Cabo AND Verde OR Cameroon OR Central AND African AND Republic OR Chad OR Comoros OR Congo, AND Democratic AND Republic AND of OR Congo, AND Republic AND of OR Cote AND D'ivoire OR Equatorial AND Guinea OR Eritrea OR Eswatini OR Ethiopia OR Gabon OR Gambia OR Ghana OR Guinea OR Guinea-Bissau OR Kenya OR Lesotho OR Liberia OR Madagascar OR Malawi OR Mali OR Mauritania OR Mauritius OR Mozambique OR Namibia OR Niger OR Nigeria OR Rwanda OR Sao AND Tome AND Principe OR Senegal OR Seychelles OR Sierra AND Leone OR Somalia OR South AND Africa OR South AND Sudan OR Sudan OR Tanzania OR Togo OR Uganda OR Zambia OR Zimbabwe ) *Limiters X Publication Date: 20130101-20231231* | **12** |
| Scopus | ( ( ( TITLE-ABS-KEY ( ''point AND prevalence'' ) OR TITLE-ABS-KEY ( ''point AND prevalence AND survey'' ) ) ) AND ( ( TITLE-ABS-KEY ( ''antibiotic AND use'' ) OR TITLE-ABS-KEY ( ''antimicrobial AND use'' ) OR TITLE-ABS-KEY ( "antimicrobial stewardship" ) OR TITLE-ABS-KEY ( "antimicrobial stewardship program" ) OR TITLE-ABS-KEY ( "rational antimicrobial use" ) ) ) ) AND ( TITLE-ABS-KEY ( angola OR benin OR botswana OR burkina AND faso OR burundi OR cabo AND verde OR cameroon OR central AND african AND republic OR chad OR comoros OR congo, AND democratic AND republic AND of OR congo, AND republic AND of OR cote AND d'ivoire OR equatorial AND guinea OR eritrea OR swaziland OR ethiopia OR gabon OR gambia OR ghana OR guinea OR guinea-bissau OR kenya OR lesotho OR liberia OR madagascar OR malawi OR mali OR mauritania OR mauritius OR mozambique OR namibia OR niger OR nigeria OR rwanda OR sao AND tome AND principe OR senegal OR seychelles OR sierra AND leone OR somalia OR south AND africa OR south AND sudan OR sudan OR tanzania OR togo OR uganda OR zambia OR zimbabwe ) ) AND *PUBYEAR > 2013 AND PUBYEAR < 2024* | **56** |
| Web of Science | ((TS=(point prevalence* OR point prevalence survey*)) AND TS=(antibiotic use* OR antimicrobial use* OR antimicrobial stewardship* OR antimicrobial stewardship program* OR rational antimicrobial use*)) AND TS=(Angola OR Benin OR Botswana OR Burkina AND faso OR Burundi OR Cabo AND Verde OR Cameroon OR Central AND African AND Republic OR Chad OR Comoros OR Congo, AND Democratic AND Republic AND of OR Congo, AND Republic AND of OR Cote AND D'ivoire OR Equatorial AND Guinea OR Eritrea OR Eswatini OR Ethiopia OR Gabon OR Gambia OR Ghana OR Guinea OR Guinea-Bissau OR Kenya OR Lesotho OR Liberia OR Madagascar OR Malawi OR Mali OR Mauritania OR Mauritius OR Mozambique OR Namibia OR Niger OR Nigeria OR Rwanda OR Sao AND Tome AND Principe OR Senegal OR Seychelles OR Sierra AND Leone OR Somalia OR South AND Africa OR South AND Sudan OR Sudan OR Tanzania OR Togo OR Uganda OR Zambia OR Zimbabwe) and 2013 or 2014 or 2015 or 2016 or 2017 or 2018 or 2019 or 2020 or 2021 or 2022 or 2023 or 2024 | **155** |

**Appendix II: PRISMA 2020 Checklist**

| **Section and Topic** | **Item #** | **Checklist item** | **Location where item is reported** |
| --- | --- | --- | --- |
| **TITLE** | | |  |
| Title | 1 | Identify the report as a systematic review. | Page 1 |
| **ABSTRACT** | | |  |
| Abstract | 2 | See the PRISMA 2020 for Abstracts checklist. | Page 2 |
| **INTRODUCTION** | | |  |
| Rationale | 3 | Describe the rationale for the review in the context of existing knowledge. | Page 3 |
| Objectives | 4 | Provide an explicit statement of the objective(s) or question(s) the review addresses. | Page 3 & 4 |
| **METHODS** | | |  |
| Eligibility criteria | 5 | Specify the inclusion and exclusion criteria for the review and how studies were grouped for the syntheses. | Page 4 |
| Information sources | 6 | Specify all databases, registers, websites, organisations, reference lists and other sources searched or consulted to identify studies. Specify the date when each source was last searched or consulted. | Page 4 |
| Search strategy | 7 | Present the full search strategies for all databases, registers and websites, including any filters and limits used. | Page 4 |
| Selection process | 8 | Specify the methods used to decide whether a study met the inclusion criteria of the review, including how many reviewers screened each record and each report retrieved, whether they worked independently, and if applicable, details of automation tools used in the process. | Page 4&5 |
| Data collection process | 9 | Specify the methods used to collect data from reports, including how many reviewers collected data from each report, whether they worked independently, any processes for obtaining or confirming data from study investigators, and if applicable, details of automation tools used in the process. | Page 5 |
| Data items | 10a | List and define all outcomes for which data were sought. Specify whether all results that were compatible with each outcome domain in each study were sought (e.g. for all measures, time points, analyses), and if not, the methods used to decide which results to collect. | Page 5 |
|  | 10b | List and define all other variables for which data were sought (e.g. participant and intervention characteristics, funding sources). Describe any assumptions made about any missing or unclear information. | Page 6 |
| Study risk of bias assessment | 11 | Specify the methods used to assess risk of bias in the included studies, including details of the tool(s) used, how many reviewers assessed each study and whether they worked independently, and if applicable, details of automation tools used in the process. | Page 6 |
| Effect measures | 12 | Specify for each outcome the effect measure(s) (e.g. risk ratio, mean difference) used in the synthesis or presentation of results. | Page 6 |
| Synthesis methods | 13a | Describe the processes used to decide which studies were eligible for each synthesis (e.g. tabulating the study intervention characteristics and comparing against the planned groups for each synthesis (item #5)). | Page 6 |
|  | 13b | Describe any methods required to prepare the data for presentation or synthesis, such as handling of missing summary statistics, or data conversions. | Page 6 |
|  | 13c | Describe any methods used to tabulate or visually display results of individual studies and syntheses. | Page 6 |
|  | 13d | Describe any methods used to synthesize results and provide a rationale for the choice(s). If meta-analysis was performed, describe the model(s), method(s) to identify the presence and extent of statistical heterogeneity, and software package(s) used. | Page 6 |
|  | 13e | Describe any methods used to explore possible causes of heterogeneity among study results (e.g. subgroup analysis, meta-regression). | Page 6 |
|  | 13f | Describe any sensitivity analyses conducted to assess robustness of the synthesized results. | Page 6 |
| Reporting bias assessment | 14 | Describe any methods used to assess risk of bias due to missing results in a synthesis (arising from reporting biases). | Page 5 |
| Certainty assessment | 15 | Describe any methods used to assess certainty (or confidence) in the body of evidence for an outcome. | Not applicable |
| **RESULTS** | | |  |
| Study selection | 16a | Describe the results of the search and selection process, from the number of records identified in the search to the number of studies included in the review, ideally using a flow diagram. | Page 6&7 |
|  | 16b | Cite studies that might appear to meet the inclusion criteria, but which were excluded, and explain why they were excluded. | Page 6&7 |
| Study characteristics | 17 | Cite each included study and present its characteristics. | Page 6&7 |
| Risk of bias in studies | 18 | Present assessments of risk of bias for each included study. | Page 6 (Table 1) |
| Results of individual studies | 19 | For all outcomes, present, for each study: (a) summary statistics for each group (where appropriate) and (b) an effect estimate and its precision (e.g. confidence/credible interval), ideally using structured tables or plots. | Not applicable |
| Results of syntheses | 20a | For each synthesis, briefly summarise the characteristics and risk of bias among contributing studies. | Page 7&8 |
|  | 20b | Present results of all statistical syntheses conducted. If meta-analysis was done, present for each the summary estimate and its precision (e.g. confidence/credible interval) and measures of statistical heterogeneity. If comparing groups, describe the direction of the effect. | Page 7&8 |
|  | 20c | Present results of all investigations of possible causes of heterogeneity among study results. | Page 7&8 |
|  | 20d | Present results of all sensitivity analyses conducted to assess the robustness of the synthesized results. | Page 7&8 |
| Reporting biases | 21 | Present assessments of risk of bias due to missing results (arising from reporting biases) for each synthesis assessed. | Page 7&8 |
| Certainty of evidence | 22 | Present assessments of certainty (or confidence) in the body of evidence for each outcome assessed. | Not applicable |
| **DISCUSSION** | | |  |
| Discussion | 23a | Provide a general interpretation of the results in the context of other evidence. | Page 8&9 |
|  | 23b | Discuss any limitations of the evidence included in the review. | Page 8&9 |
|  | 23c | Discuss any limitations of the review processes used. | Page 9 |
|  | 23d | Discuss implications of the results for practice, policy, and future research. | Page 10 |
| **OTHER INFORMATION** | | |  |
| Registration and protocol | 24a | Provide registration information for the review, including register name and registration number, or state that the review was not registered. | Page 4 |
|  | 24b | Indicate where the review protocol can be accessed, or state that a protocol was not prepared. | Page 4 |
|  | 24c | Describe and explain any amendments to information provided at registration or in the protocol. | Not applicable |
| Support | 25 | Describe sources of financial or non-financial support for the review, and the role of the funders or sponsors in the review. | Page 4 |
| Competing interests | 26 | Declare any competing interests of review authors. | Page 10 |
| Availability of data, code and other materials | 27 | Report which of the following are publicly available and where they can be found: template data collection forms; data extracted from included studies; data used for all analyses; analytic code; any other materials used in the review. | Page 10 |

Appendix III Risk of bias assessment for 32 eligible studies

| **Author and country** | **Q1** | **Q2** | **Q3** | **Q4** | **Q5** | **Q6** | **Q7** | **Q8** | **Q9** | **RoB Score** |
| --- | --- | --- | --- | --- | --- | --- | --- | --- | --- | --- |
| Usman et al (2020) | Y | Y | Y | Y | Y | Y | Y | Y | Y | 9 |
| Umeokonkwo et al. (2019) | Y | Y | Y | Y | Y | Y | N | Y | Y | 8 |
| Manga et al. (2021) | Y | Y | U | Y | N | Y | Y | Y | Y | 7 |
| Aboderin et al. (2021) | Y | Y | Y | Y | Y | Y | Y | Y | Y | 9 |
| Nsofor et al. (2016) | Y | N | Y | Y | Y | Y | Y | Y | Y | 8 |
| Fowotade et al. (2020) | Y | Y | Y | Y | Y | Y | Y | Y | Y | 9 |
| Nnadozie et al. (2021) | Y | Y | Y | Y | Y | Y | Y | Y | N | 8 |
| Oduyebo et al. (2017) | Y | U | Y | Y | Y | Y | Y | Y | Y | 8 |
| Ogunleye et al. (2022) | Y | Y | Y | U | Y | Y | Y | Y | Y | 8 |
| Labi et al. (2018) | Y | Y | Y | Y | Y | Y | Y | Y | Y | 9 |
| Labi et al. (2021) | U | Y | Y | Y | Y | Y | Y | Y | U | 7 |
| Labi et al. (2018) | Y | Y | Y | U | Y | Y | Y | Y | Y | 8 |
| Amponsah et al. (2021) | Y | Y | Y | Y | Y | Y | Y | Y | Y | 9 |
| Bediako-B et al. (2019) | Y | Y | Y | Y | Y | U | Y | N | Y | 7 |
| Daniel Ankrah et al. (2021) | Y | Y | Y | Y | Y | Y | Y | Y | Y | 9 |
| Kamita et al. (2022) | Y | Y | Y | Y | Y | Y | Y | Y | Y | 9 |
| Momanyi et al. (2019) | Y | Y | Y | Y | Y | Y | Y | Y | Y | 9 |
| Okoth et al. (2018) | Y | Y | U | Y | Y | Y | Y | Y | Y | 8 |
| Omulo et al. (2022) | Y | Y | Y | Y | Y | Y | Y | Y | Y | 9 |
| Skosana et al. (2021) | Y | Y | Y | Y | Y | Y | Y | Y | Y | 9 |
| Skosana et al. (2021) | Y | Y | Y | Y | Y | Y | Y | Y | Y | 9 |
| Horumpende et al. (2020) | Y | Y | Y | Y | Y | Y | Y | Y | Y | 9 |
| Seni et al. (2020) | Y | Y | Y | Y | U | Y | Y | Y | Y | 9 |
| Bunduki et al. (2021) | Y | Y | Y | Y | Y | Y | Y | Y | Y | 9 |
| Fentie et al. (2022) | Y | Y | Y | Y | Y | Y | Y | Y | Y | 9 |
| Kiggundu et al. (2022) | Y | Y | Y | Y | Y | Y | Y | Y | Y | 9 |
| Ahoyo et al. (2012) | Y | Y | Y | N | Y | Y | Y | Y | U | 7 |
| BDA Paramadhas et al.(2019) | Y | Y | Y | Y | Y | Y | Y | Y | Y | 9 |
| Daniel Ankrah (2021)  Excluded | Y | N | Y | Y | N | N | Y | N | N | 4 |
| Cornelius C. Dodoo (2021)  Excluded | Y | Y | N | Y | N | Y | N | N | Y | 5 |
| Anthony Enimil (2022)  Excluded | Y | N | N | N | Y | Y | Y | N | N | 4 |
| Nwajiobi-Princewill (2021)  Excluded | Y | Y | Y | N | N | N | N | N | Y | 4 |

Total Yes (Y) means studies that fulfilled at least seven from the domain of nine criteria = 94%

Total No (N) means studies with less than seven domains that did not answer from the domain of nine criteria = 2.5%

Ttotal Unclear (U) means studies with less than seven domains that are unclear to answer from the domain of nine criteria = 3.5%

Overall risk of bias assessment score (237/252) = 94%

Remark: The risk of bias for each eligible study is calculated from the domain of nine criteria

**Appendix IV:** **Funnel plot showing symmetric distribution of studies on point prevalence of evidence-based use of antibiotics among hospitalized patients in sub-Saharan Africa**


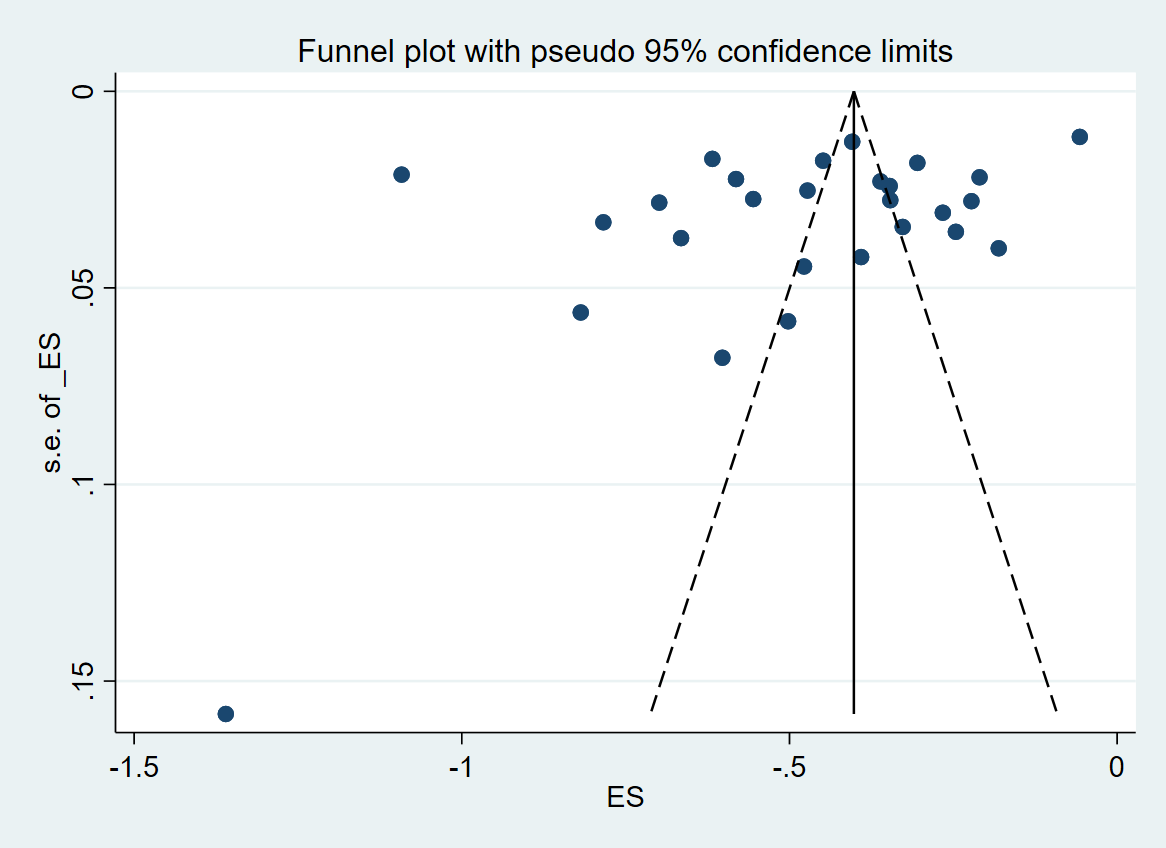

Supplement: Supplementary file 1 — Supplementary Information. [file 41598_2024_62651_MOESM1_ESM.docx]
